# Src in endosomal membranes promotes exosome secretion and tumor progression

**DOI:** 10.1038/s41598-019-39882-z

**Published:** 2019-03-01

**Authors:** Tomoya Hikita, Atsushi Kuwahara, Risayo Watanabe, Mamiko Miyata, Chitose Oneyama

**Affiliations:** 1 Division of Cancer Cell Regulation, Aichi Cancer Center Research Institute, Nagoya, Japan; 2 Department of Oncogene Research, Research Institute for Microbial Diseases, Osaka University, Suita, Osaka Japan; 3 JST, PRESTO, Nagoya, Japan

## Abstract

c-Src is a membrane-associated tyrosine kinase that has key roles in the signaling transduction that controls cell growth, adhesion, and migration. In the early stage of carcinogenesis, c-Src is activated under the plasma membrane and transduces oncogenic signals. Here we show that c-Src localized to the endosomal membrane has unique functions in c-Src–transformed cells. Our results indicate that activated c-Src in the endosomal membrane promoted the secretion of exosomes, in which c-Src was encapsulated. In addition, the ESCRT-interacting molecule, Alix was identified as a c-Src–interacting protein in exosomes. We revealed that the interaction between the SH3 domain of c-Src and the proline-rich region of Alix activates ESCRT–mediated intra-luminal vesicle (ILV) formation, resulting in the upregulation of exosome secretion in c-Src–transformed cells. We observed also a correlation between malignant phenotypes and Alix–dependent aberrant exosome secretion in Src–upregulated cancer cells. Collectively, our findings provide a unique mechanism for the upregulation of exosomes in cancer cells, as well as new insights into the significance of exosome secretion in cancer progression.

## Introduction

*c-src* is the first identified proto-oncogene and its product is a membrane-associated non-receptor type tyrosine kinase^1,2^. Studies have shown that c-Src plays critical roles in signal transduction related to cellular survival, proliferation, and motility^3–5^. In addition, the expression and activity of c-Src is frequently enhanced in various human cancers, suggesting it plays a role in cancer development^6–8^. However, mutation of the *c-src* gene is rarely observed in tumor tissue^9,10^. In normal cells, the activity of c-Src is strictly controlled by Csk, and it has been suggested that the breakdown of the c-Src regulatory system may lead to cancer development^11,12^. It is known that c-Src associates with the plasma membrane via myristoylation in order to transmit signals from the outside to the inside of cells^2^. Evidence from previous studies, including our own, has suggested that c-Src is activated under the plasma membrane in the early stage of carcinogenesis and transmits oncogenic signals^13^. On the other hand, it has also been reported that c-Src localizes and functions not only in the plasma membrane, but also in the inner membrane including endosomal membrane^14,15^. However, while some reports have investigated the regulation of its localization, the functional significance of endosomal c-Src in cancer is not well understood.

Exosomes are extracellular membrane vesicles that are believed to be derived from endosomes and thought to be responsible for intercellular communication^5,16^. Indeed, information can be transferred between cells by molecules such as proteins, lipids, and miRNAs in exosomes^17,18^. Exosomes are secreted by various cells, including cancer cells, to regulate the local microscopic environment^19,20^. In addition, exosomes can be transmitted to distant sites via the bloodstream where they may contribute to premetastatic niche formation^20,21^. These findings strongly suggest that exosomes are important for cancer development. Since the amount and content of exosomes changes in cancer, liquid biopsies that use exosomes for cancer diagnosis have been attracting increasing attention^22^. However, there remains a number of unresolved questions regarding how exosomes are formed from endosomes and where their cargo is loaded and secreted^23^. Moreover, the mechanisms by which they change and the biological importance of exosome upregulation in cancer remains elusive^24^.

In this study, we first examined the localization of activated c-Src using Csk^−/−^ cells, which are turned cancerous by Src activation^13^, and found that c-Src localized to not only focal adhesion, but also endosomal membranes. Such cells showed an increased secretion of exosomes in which activated Src molecules were encapsulated. In order to analyze the role of c-Src in exosome formation, we then searched molecules that bind to the activated c-Src within exosomes. We identified Alix, which is known to interact with several ESCRT (endosomal sorting complex required for transport) proteins including Tsg101 and CHMP4, and thought to be involved in the formation of intra-luminal vesicles (ILV)^25,26^. Although Alix is used as a canonical exosome marker, as well as a marker of CD9 or CD63 in exosomes derived from different cell types, the mechanisms underlying the regulation of its function and precise role in cancer cells are not well known. In this study, our findings indicated that the interaction between the SH3 domain of c-Src and the proline-rich region (PRR) of Alix activates ESCRT-mediated ILV formation. We observed this phenomenon also in Src-upregulated human cancer cells and found a correlation between cancer phenotypes and Alix–dependent aberrant exosome secretion. Interestingly, the inhibition of exosome secretion, observed not only with the shRNA of Alix but also the shRNA of Rab27b and an inhibitor of sphingomyelinase (GW4869), suppressed cancer phenotypes of exosome–secreting cells, suggesting that appropriate secretion of exosomes contributes to the maintenance of cancer phenotypes. Collectively, our results provide a novel mechanism for the upregulation of exosomes in cancer cells and new insights for its significance in cancer progression.

## Results

### Active c-Src localizes to late-endosome membranes and promotes exosome secretion

**Figure 1:**
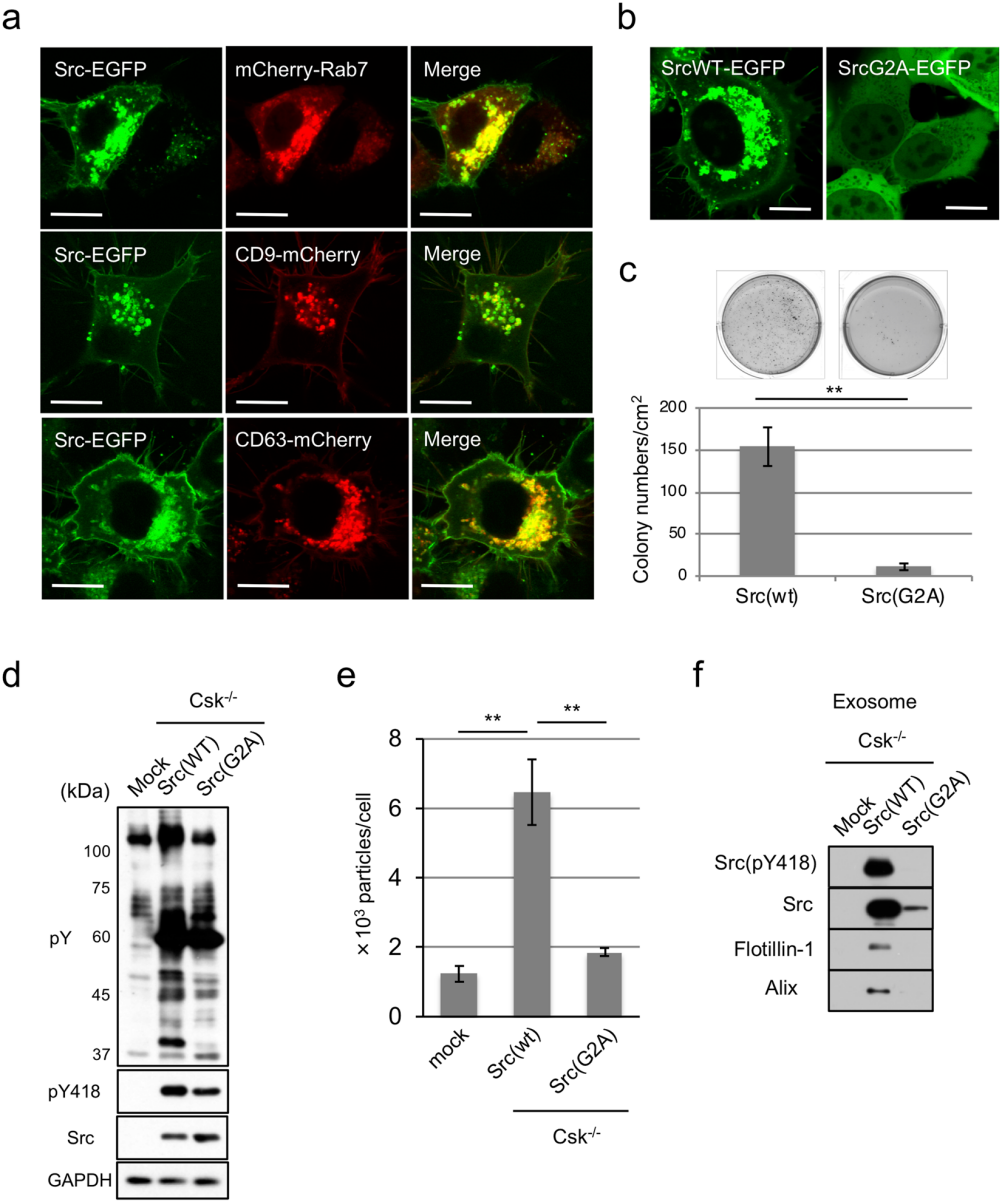
Active Src in the late endosome membrane increased exosome secretion in Src-induced transformation. (**a**) EGFP-conjugated Src (Src-EGFP) and mCherry-conjugated Rab7, CD9, or CD63, were expressed in Csk^−/−^ cells, and the localization of Src was analyzed. Scale bar = 10 µm. (**b**) EGFP-conjugated wild-type (SrcWT-EGFP) or membrane-anchorage-deficient Src (SrcG2A-EGFP) was expressed in Csk^−/−^ cells, and the localization of Src was analyzed. Scale bar = 10 µm. (**c**) Soft-agar colony-formation assays of cells indicated in (**b**) for 6 days. Representative dishes (upper panels) and average colonies/cm^2^ (lower panels) obtained from three independent experiments were shown. **p < 0.01, by Student’s *t*-test against Src(wt). (**d**) The whole-cell lysates of parental Csk^−/−^ cells (mock) and cells indicated in (**b**) were analyzed by immunoblotting with the indicated antibodies. (**e**) Cells used in (**d**) were subjected to exosome preparation followed by NTA for quantitative measurement of isolated exosome particles. **p < 0.01, by ANOVA with Dunnett’s post hoc test. (**f**) The exosome lysates were immunoblotted with the indicated antibodies, including canonical exosome markers. Relative values were obtained from three independent assays (**c**,**e**). For all graphs, error bars indicate the mean ± SD of three independent measurements. Uncropped gel images for panels d and f are shown in Supplementary Fig. 6.

To analyze the implication of the spatial localization and transforming ability of Src, we developed a model experimental system using Csk^−/−^ cells that express c-Src conjugated with EGFP. In this system, exogenous expression of c-Src efficiently induced cell transformation in a Csk–dependent manner^13^. We next examined the intracellular localization of active c-Src. By focusing on the adhesion surface, active c-Src localized to focal adhesions in Src–transformed cells, as previously reported^13,27^ (SupFig. 1a, upper panel). However, by focusing on the intermediate section, we observed that primarily active c-Src localized to the intracellular perinuclear region (SupFig. 1a, lower panel). We then examined the co-localization of active c-Src with known organelle markers, including Rab5 (early endosome), Rab7 (late endosome), and Rab11 (recycling endosome)^28,29^ (SupFig. 1b). In Src–transformed cells, c-Src co-localized with Rab5 and Rab7, but not with Rab11, which resulted in active c-Src localizing to early/late endosomes vesicles (Fig. 1a and SupFig. 1b). These observations led us to further examine the co-localization of c-Src with late endosome markers such as CD9 and CD63, as well as canonical exosome markers^30^. As expected, c-Src co-localized with CD9 and CD63 in Src–transformed cells (Fig. 1a). To confirm the localization of exogenous c-Src itself, we performed immunostaining using anti-Src or anti-CD63 antibodies. In Src-expressing cells, c-Src and CD63 were localized to the membranes of endosomes, confirming the endosome localization of c-Src in Src–transformed cells (SupFig. 1b,c). We also observed that the level of c-Src expression in Csk^−/−^ cells (Src-mScarlet#4 or #9) was similar to that observed in Csk^+/+^ cells, which cannot be transformed, confirming that the localization of c-Src in the endosome membrane is not due to Src overexpression (SupFig. 1d). These findings suggest that c-Src intrinsically is localized to the endosome membrane as well as the plasma membrane.

We further addressed the relevance of the localization of active c-Src to the endosome membrane with transforming ability. To this end, we developed a Gly2 to Ala substitution (G2A) that mutates a certain myristoylation site, causing Src to be diffused into cells (Fig. 1b) and colony-forming activity to be lost^31^ (Fig. 1c). We found that Src kinase activity was not affected by localization, as indicated by the retention of autophosphorylation at pY418 (Fig. 1d); however, the G2A mutation inhibited the activation of downstream components, as indicated by phosphorylated proteins (pY; Fig. 1d). These observations appear to demonstrate that c-Src transduces oncogenic signaling through the membranes. Since the majority of the population of active c-Src in Csk^−/−^ cells was localized to the late endosome, we examined the effect of active c-Src localization on the secretion of endosome-derived exosomes. We prepared the exosomes by ultracentrifugation; extracellular vesicles in Src–transformed Csk^−/−^ cells were confirmed to be exosomes based on nanoparticle tracking analysis (NTA) and Western blotting analysis of canonical exosome markers such as flotillin-1 and Alix (Fig. 1e,f). Consistent with recent reports^32^, exosome secretion was significantly increased with Src activation (Fig. 1e). Interestingly, active c-Src localized to endosome membranes served to increase exosome secretion, but c-Src diffused intracellularly did not, indicating that these observations were accompanied with the transforming activity of active c-Src (Fig. 1c,e). Furthermore, we observed that active c-Src was encapsulated in exosomes secreted from Src–transformed cells (Fig. 1f). These observations raise the possibility that active c-Src localized to endosome membranes induces exosome secretion.

### Exosome secretion induced by c-Src and v-Src

**Figure 2:**
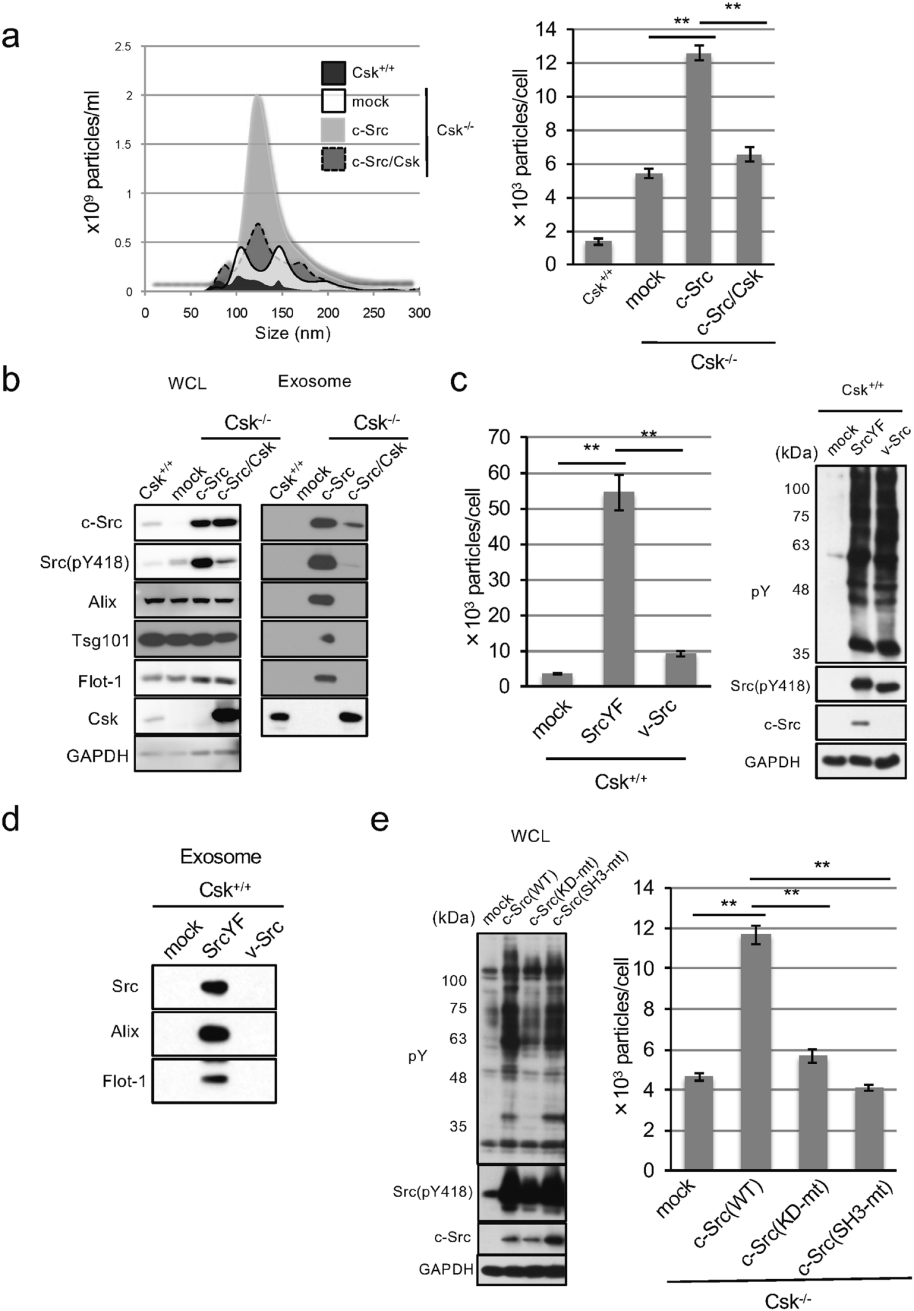
Src promotes exosome secretion via its kinase activity and SH3-mediated interaction. (**a**) NTA analysis for the quantitative measurement of isolated exosome particles from Csk^+/+^ cells and Csk^−/−^ cells expressing mock, c-Src, or c-Src with Csk (raw data; left panel, the number of particles; right panel). **p < 0.01, by ANOVA with Dunnett’s post hoc test. (**b**) The whole-cell lysates (WCL; left panels) or exosome lysates (Exosome; right panels) from cells indicated in (**a**) were immunoblotted with the indicated antibodies, including canonical exosome markers. (**c**) Csk^+/+^ cells were expressed with mock, constitutively active c-Src (SrcYF), or v-Src, and whole cell lysates were immunoblotted with the indicated antibodies (right panels). NTA analysis for the quantitative measurement of isolated exosome particles from each cell (left panel). **p < 0.01, ANOVA with Dunnett’s post hoc test. (**d**) Exosome lysates from cells indicated in (**c**) were immunoblotted with the indicated antibodies, including canonical exosome markers. (**e**) Csk^−/−^ cells expressing mock, wild-type (WT), kinase-dead (KD-mt), or SH3-mutant (SH3-mt) of c-Src were subjected to immunoblotting with the indicated antibodies (left panels) and NTA analysis for the quantitative measurement of isolated exosome particles from each cell (right panel). **p < 0.01, ANOVA with Dunnett’s post hoc test. For all graphs, error bars indicate the mean ± SD of three independent measurements. Uncropped gel images for panels b, c, d and e are shown in Supplementary Fig. 6.

We next addressed the molecular mechanisms underlying endosomal Src–mediated exosome secretion, which is accompanied with Src–mediated transformation. Previously, we developed an experimental system using Csk^−/−^ cells in which exogenous expression of c-Src, at levels of more than twice the endogenous c-Src, could efficiently induce cell transformation in a Csk–dependent manner^13^ (SupFig. 2a). Using these cells, we compared the level of exosome secretion between non-transformed cells (Csk^+/+^ cells, Csk^−/−^ cells, and Csk^−/−^ cells expressing exogenous c-Src and Csk) and c-Src–transformed cells (Csk^−/−^ cells expressing c-Src) by NTA analysis. The size population of exosomes was slightly larger in c-Src–transformed cells than non-transformed cells (Fig. 2a). In addition, exosome secretion was substantially increased with c-Src transformation, as judged by NTA and Western blotting analyses of exosome markers such as Alix, Tsg101, and Flotillin-1 (Fig. 2a, right panel and 2b). Interestingly, we found that active c-Src (Src pY418) was significantly concentrated in exosomes from c-Src–transformed cells (Fig. 2b, compare left panels and right panels) with several phosphorylated proteins including AnnexinA2, a marker of exosome and substrate of Src (SupFig. 2b). These observations suggest a potential role of c-Src in promoting exosome secretion and transformation via its kinase activity. We then examined exosome secretion by both an oncoviral v-Src and a constitutively active c-Src mutant with a Tyr to Phe substitution at Tyr529 (SrcYF) in Csk^+/+^ cells, which showed significant transformation^13^. Unexpectedly, we found that SrcYF induced exosome secretion; however, v-Src failed to induce exosome secretion (Fig. 2c,d). Accompanied with this observation, SrcYF but not v-Src was found to be concentrated in the exosomes (Fig. 2d). Since v-Src has several mutations in the SH3 domain compared with SrcYF, to confirm the contribution of the SH3 domain in the increased exosome secretion, we developed SrcSH3-mt, a mutant of c-Src with the Trp118 to Ala substitution that is deficient for SH3–mediated protein-protein interactions. This mutation did not alter the endosomal distribution of Src (SupFig. 2c). As expected, the c-Src mutant, as well as the kinase-dead mutant of Src (SrcKD-mt), failed to promote exosome secretion, which is consistent with the contribution of the kinase activity of c-Src in Csk^−/−^ cells (Fig. 2a,b,e). Under these conditions, exosome secretion ability appears to be correlated with the transformation of Csk^−/−^ cells (SupFig. 2d). Taken together, these findings suggest that SH3 domain–mediated proteins interacting with c-Src are required for the promotion of exosome secretion and they are buried in the exosome.

### Interaction between c-Src and Alix

**Figure 3:**
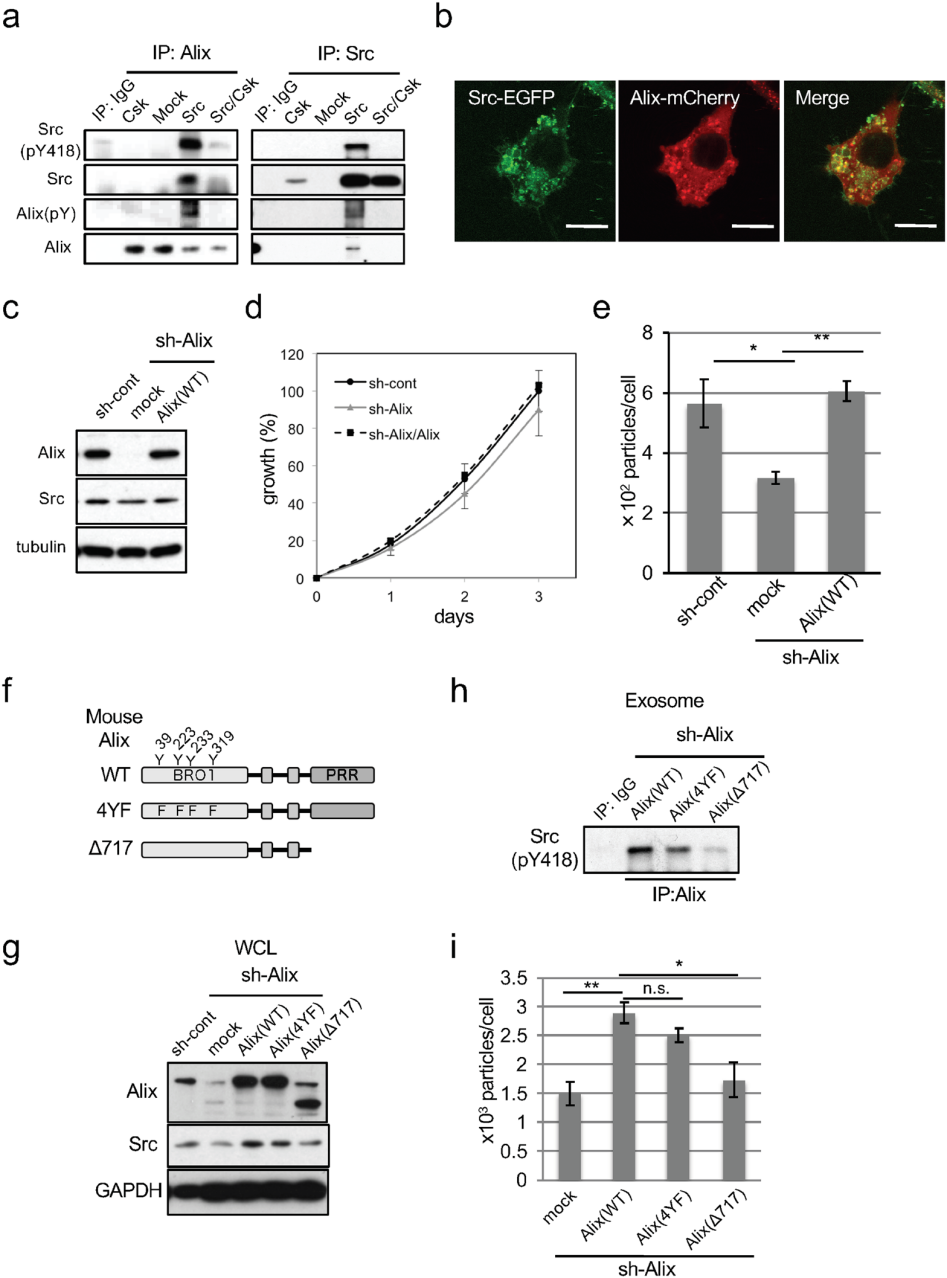
Alix is required for Src-induced exosome upregulation through its interaction with Src. (**a**) Cell lysates from Csk^−/−^ cells expressing mock, Csk, c-Src, or c-Src with Csk were subjected to immunoprecipitation (IP) with Alix or Src, followed by immunoblotting with the indicated antibodies. (**b**) Intracellular localization of Src (green) and Alix (red) in Csk^−/−^ cells expressing Src-EGFP and Alix-mCherry were analyzed. Scale bar = 10 µm. (**c**) Total cell lysates from c-Src-transformed Csk^−/−^ cells expressing control (sh-cont) or Alix shRNA (sh-Alix) with or without sh-resistant human Alix were analyzed by immunoblotting with the indicated antibodies. (**d**) Anchorage-dependent growth of cells indicated in panel (c) was examined by an *in vitro* proliferation assay with WST-1. (**e**) NTA analysis for the quantitative measurement of isolated exosome particles from cells indicated in panel (c). *p < 0.05 and **p < 0.01, by ANOVA with Dunnett’s post hoc test. (**f**) Schematic structures of mouse Alix. Locations of mutated amino acids are indicated. BRO1: BRO1 domain, PRR: proline-rich region. (**g**) Alix mutant constructs indicated in (**f**) were expressed in Src-transformed Csk^−/−^ cells transfected with Alix shRNA (sh-Alix). Total cell lysates were analyzed by immunoblotting with the indicated antibodies. (**h**) Exosome lysates from cells indicated in (**g**) were immunoprecipitated with anti-Alix and subjected to immunoblotting with active Src (Src pY418). (**i**) NTA analysis for the quantitative measurement of isolated exosome particles from cells indicated in panel (g). *p < 0.05 and **p < 0.01, by ANOVA with Dunnett’s post hoc test. *n.s*., not significant. Error bars indicate the mean ± SD of three independent measurements. Uncropped gel images for panels a, c, g and h are shown in Supplementary Fig. 6.

To further elucidate the mechanism of c-Src-induced exosome secretion, we searched for c-Src binding proteins in the exosomes secreted from c-Src–transformed cells using the Flag-tagged c-Src protein. Liquid chromatography-tandem mass spectrometry (LC-MS/MS) analysis revealed that the co-immunoprecipitated fraction with c-Src included Alix (also known as PDCD6IP), which is reported to be phosphorylated by Src and has several repeats of proline-rich-regions (PRRs) in the C-terminus region^26^ (Fig. 3f). We next performed immunoprecipitation assays and confirmed that Alix indeed interacts with c-Src in Src-transformed cells, but not in non-transformed Csk^−/−^ cells (Fig. 3a). We also observed that Alix bound to c-Src in exosomes was tyrosine phosphorylated (Fig. 3a; WB with Alix(pY)). We further examined whether c-Src co-localized with Alix in Src-transformed cells, and found that a subpopulation of Alix co-localized with active Src in endosome membranes (Fig. 3b). These observations suggest that the interaction of active c-Src and Alix in the endosome triggers endosome–derived exosome formation.

We then performed knockdown experiments to evaluate the importance of Alix in c-Src–induced exosome upregulation. An *in vitro* proliferation assay showed that Alix- knockdown did not suppress anchorage-dependent growth of these cells (Fig. 3c,d). However, knockdown of Alix significantly suppressed c-Src–induced exosome secretion. Moreover, rescue experiments using the expression of shRNA-resistant Alix in Alix-knockdown cells showed the restoration of exosome secretion in c-Src–transformed cells (Fig. 3c,e). To elucidate the details of this c-Src–Alix interaction, we next examined the effects of two Alix mutants on exosome secretion. One mutant, Δ717, lacks proline-rich regions (PRRs) that would be required for SH3–mediated interaction and the other, 4YF, has four phenylalanines at Tyr residues that found to be phosphorylated in the exosome by LC-MS/MS analysis (Fig. 3f). As shown in Fig. 3g,h, although the 4YF mutant in exosomes interacted with active c-Src, the deletion mutant lacking PRR (Δ717) failed to interact with active c-Src. These results demonstrate that the specific interaction between c-Src (SH3 domain) and Alix (PRR) is critical for exosome secretion in c-Src–transformed cells. Accompanied with the c-Src-Alix interaction, exosome secretion suppressed by Alix knockdown was restored with the expression of Alix (WT) or Alix (4YF) but not of Alix (Δ717) (Fig. 3i). Taken together, these findings suggest that active c-Src interacts with Alix in endosome membranes and promotes exosome secretion.

### The c-Src–Alix interaction promotes ILV formation in MVB

**Figure 4:**
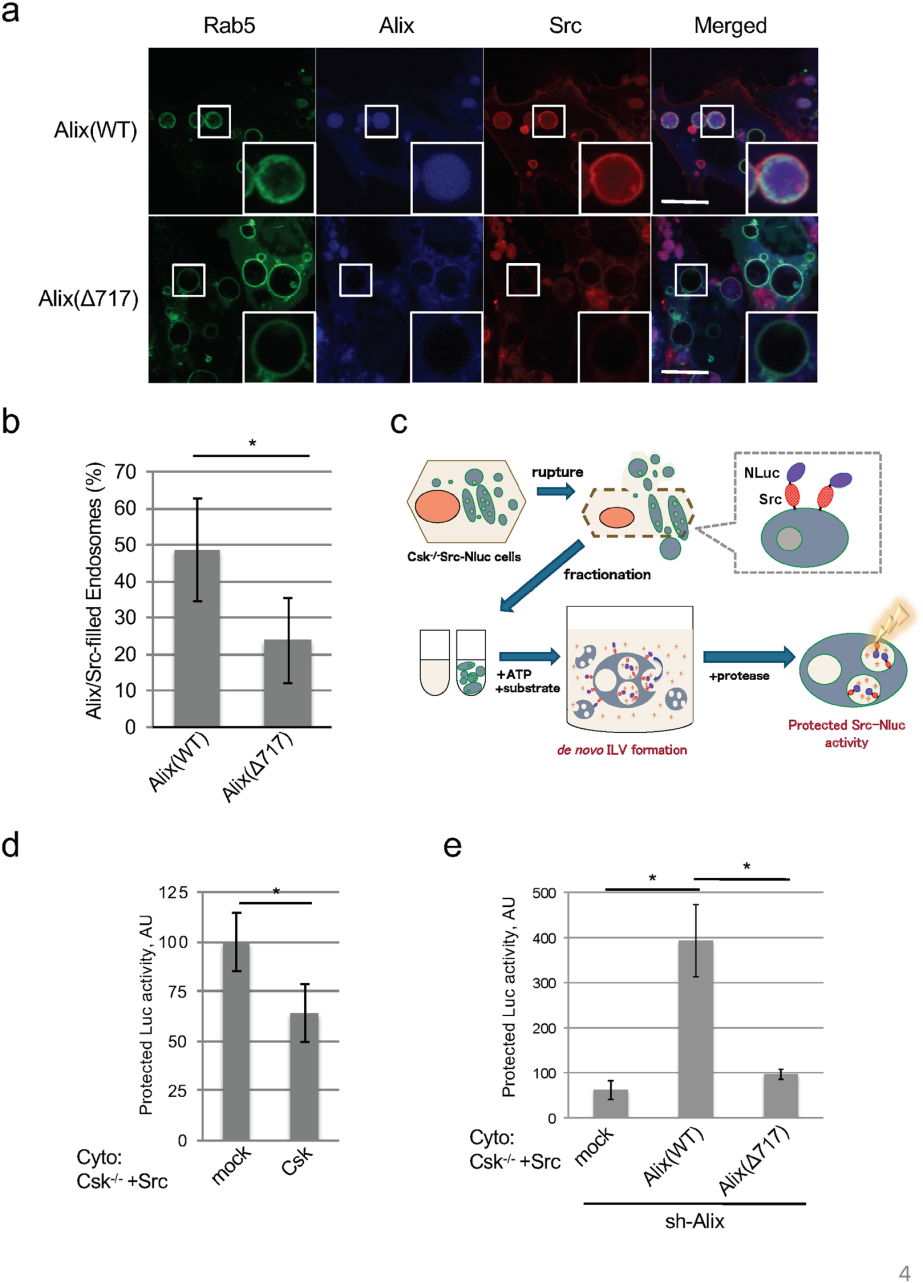
Interaction of Src with Alix enhances ILV formation in multivesicular body formation. (**a**) Localization of Src and Alix in enlarged endosomes. Csk^−/−^ cells expressing Src-mScarlet and wild-type (WT) or PRR-deficient (Δ717) of the Alix-Halo-tag were co-transfected with Rab5 (Q79L)-GFP, added to the Cyan-Halo-tag ligand, and imaged by confocal microscopy. Experiments were conducted three times with similar results. Scale bar = 10 µm. (**b**) Quantification of both of Alix and Src-filled endosomes. Relative percentage of Rab5 (Q79L)-GFP endosomes that was filled with Alix-tag and Src-mScarlet in cells indicated in (**a**) for 48 h were shown. *p < 0.05, by Student’s *t*-test against Alix(WT). (**c**) Schematics of the assay procedure of *in vitro* MVB formation. (**d**) c-Src activity depended *in vitro* MVB formation. The membranes from Csk^−/−^/c-Src expressing Src-Nluc and cytoplasm fraction from Csk^−/−^/c-Src expressing mock or Csk were incubated in the presence of ATP, and protected luciferase activity was measured. *p < 0.05, by Student’s *t*-test against mock. (**e**) Alix PRR region-mediated *in vitro* ILV formation in MVB. The membranes from Csk^−/−^/c-Src expressing Src-Nluc and the cytoplasm fraction from cells used in Fig. 3f were incubated with ATP, and protected luciferase activity was indicated. *p < 0.05, by ANOVA with Dunnett’s post hoc test. For all graphs, the error bars indicate the mean ± SD of three independent measurements.

Having shown that the interaction between Alix and active c-Src promotes the secretion of exosomes (in which active c-Src is encapsulated) through an Alix–dependent manner, we next examined if increased exosome secretion was caused by the promotion of ILV formation in multivesicular body (MVB). To assess the role of c-Src in Alix–dependent ILV formation, we first examined the potential co-localization of c-Src and Alix in endosomes. Src-transformed Csk^−/−^ cells were transfected with a constitutively active form of Rab5 (Q79L) to increase the fusion rate of endosomes^33^. The activation of Rab5 induced enlarged endosomes in which the co-localization of c-Src–Alix was easily distinguished by confocal immunofluorescence microscopy (GFP-Rab5: green). c-Src localized to both endosome membranes and the inner vesicles of endosomes (Src-mScarlet^34^: red). Notably, c-Src co-localized strongly with Alix (WT) in enlarged endosomes; however, there was little co-localization of c-Src and Alix (Δ717) (Alix-Halo-tag: blue; Fig. 4a,b). These results suggest that c-Src participates, together with Alix via an SH3–mediated interaction, in the formation of ILV in MVB. Further, we performed *in vitro* reconstitution assays using c-Src conjugated with luciferase (Src-Nluc) as a cargo. As illustrated in Fig. 4c, to reproduce an aspect of ILV formation in MVB and the encapsulation of Src in a cell-free reaction, we developed simple biochemical assays for the *in vitro* re-constitution of ILV formation, as described by Shurtleff *et al*.^35^. We fractionated Csk^−/−^ cells expressing c-Src-Nluc (SupFig. 3a) into endosomal membranes and cytoplasm and each fraction was incubated with a substrate of luciferase and ATP, followed by ILV/exosome formation. c-Src-Nluc in ILV was detected by incubation of the membrane and cytoplasm fractions in the presence of ATP (SupFig. 3b). Under these conditions, ILV formation was inhibited by neutralization with the anti-CHMP4 antibody, suggesting that this reaction reflected ILV/MVB formation executed by ESCRT machinery such as CHMP4 (SupFig. 3c). Further, we found that inactivation of c-Src by Csk has a suppressive effect on ILV formation (Fig. 4d). To investigate the function of Alix, we next performed rescue experiments using the cytoplasm fraction prepared from Alix-knockdown cells expressing shRNA-resistant Alix (Fig. 4e). Although ILV formation was rescued by the wild-type (WT), the PRR-deficient mutant (Δ717) in the cytoplasm fraction failed to rescue ILV formation (Fig. 4e). Together, these results suggest that the interaction of Alix and active c-Src via the PRR-SH3 domain is crucial for ILV formation and results in the promotion of exosome secretion.

### Exosome secretion in human cancer cells

**Figure 5:**
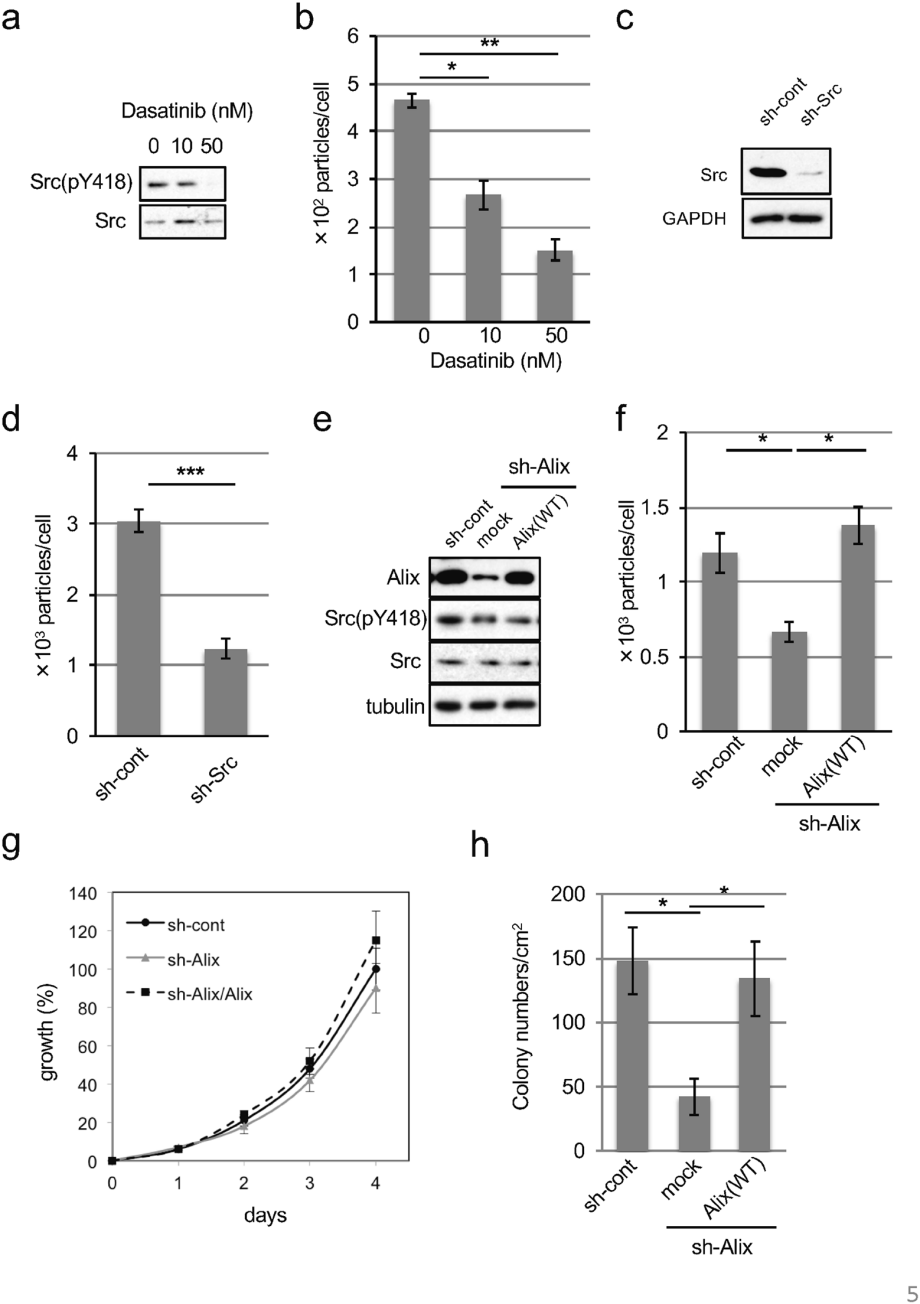
Alix promotes exosome secretion and tumor growth in human colon cancer cells harboring Src upregulation. (**a**) HT29 cells were treated with or without dasatinib at the indicated concentration for 24 hours, and the total cell lysates were immunoblotted with the antibodies indicated. (**b**) NTA analysis for the quantitative measurement of isolated exosome particles from cells indicated in panel (a). *p < 0.05 and **p < 0.01, by ANOVA with Dunnett’s post hoc test. (**c**) Total cell lysates from HT29 cells expressing control (sh-cont) or Src shRNA (sh-Src) were immunoblotted with the indicated antibodies. (**d**) NTA analysis for the quantitative measurement of isolated exosome particles from cells indicated in panel (c). ***p < 0.001, by Student’s *t*-test against sh-cont. (**e**) Total cell lysates from HT29 cells expressing control (sh-cont) or Alix shRNA (sh-Alix) with or without sh-resistant mouse Alix were immunoblotted with the indicated antibodies. (**f**) NTA analysis for the quantitative measurement of isolated exosome particles from cells indicated in panel (e). *p < 0.05, by ANOVA with Dunnett’s post hoc test. (**g**) Anchorage-dependent growth of HT29 cells indicated in panel (e). (**h**) Soft-agar colony formation assays of HT29 cells indicated in (**e**). The mean number of colonies/cm^2^ are shown. *p < 0.05, by ANOVA with Dunnett’s post hoc test. For all graphs, the error bars indicate the mean ± SD of three independent measurements. Uncropped gel images for panels a, c and e are shown in Supplementary Fig. 6.

To address the role of endogenous c-Src in human cancer, we examined the localization of Src in several human cancer cells in which c-Src is often upregulated. As shown in SupFig. 4a, c-Src was found to be localized to the endosomal membrane, and co-localized with CD63 in colon cancer cells, including HCT116 and HT29 cells. To verify active Src–Alix-mediated exosome secretion in human cancer cells, we examined the contribution of Alix and c-Src to exosome secretion in human colon cancer cells in which c-Src is upregulated. Under the conditions of Src inhibition with dasatinib, a kinase inhibitor effective for Src family kinase, which suppressed colony-forming activity in HT29 cells (Fig. 5a), exosome secretion was significantly decreased (Fig. 5b). To elucidate the role of c-Src in the secretion of exosome in human cancers, we used shRNAs to knockdown c-Src in HT29 cells and found that exosome secretion was attenuated by knockdown of Src (Fig. 5c,d). We further examined the role of Alix in human cancers and revealed that knockdown of Alix significantly suppressed exosome secretion. Rescue experiments with the expression of shRNA-resistant Alix in Alix-knockdown cells showed the restoration of exosome secretion in HT29 cells (Fig. 5e,f). Cell proliferation assay showed that Alix expression did not affect anchorage-dependent growth of these cells (Fig. 5g). In contrast, Alix knockdown potently suppressed anchorage-independent growth of HT29 cells (Fig. 5h). Conversely, the rescue of Alix increased colony growth in HT29 cells (Fig. 5h). These findings suggest that the active c-Src-Alix axis is involved in promoting exosome secretion in human cancer cells, which is accompanied by tumor growth in exosome-secreting cells. Taken together, these observations raise the possibility that exosome secretion is associated with the maintenance of anchorage-independent growth in cancer cells.

### Exosome secretion maintains tumor growth in cancer cells

**Figure 6:**
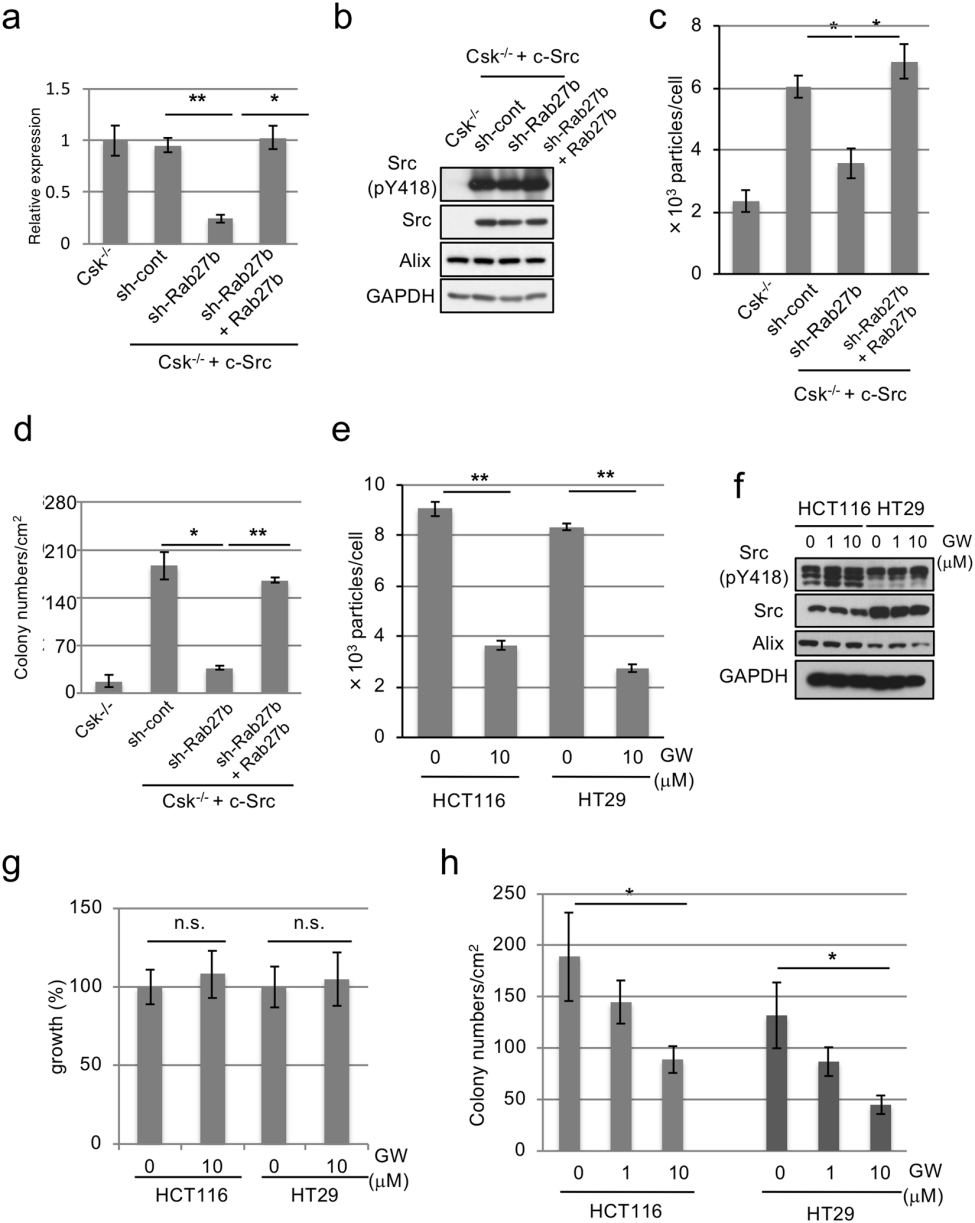
Exosome secretion promotes tumor growth of cancer cells. (**a**) Rab27b mRNA expression levels were assessed by qRT-PCR in c-Src-transformed Csk^−/−^ cells expressing control (sh-cont) or Rab27b shRNA (sh-Rab27b) with or without sh-resistant human Rab27b. *p < 0.05 and **p < 0.01, by ANOVA with Dunnett’s post hoc test. (**b**) Total cell lysates from cells in (**a**) were immunoblotted with the antibodies indicated. (**c**) NTA analysis for the quantitative measurement of isolated exosome particles from cells indicated in panel (a). *p < 0.05, by ANOVA with Dunnett’s post hoc test. (**d**) Cells indicated in (**a**) were subjected to the soft-agar colony-formation assay. The mean numbers of colonies/cm^2^ were shown. *p < 0.05 and **p < 0.01, by ANOVA with Dunnett’s post hoc test. (**e**) HCT116 cells or HT29 cells were treated with GW4869 (GW) at the indicated concentrations and NTA analysis for the quantitative measurement of isolated exosome particles are shown. **p < 0.01, by ANOVA with Dunnett’s post hoc test. (**f**) Total cell lysates from HCT116 cells or HT29 cells treated with GW4869 (GW) at the indicated concentrations for 2 days. (**g**) HCT116 cells or HT29 cells were treated with GW4869 (GW) at the indicated concentrations for 3 days and anchorage-dependent cell growth was examined with WST-1. n.s, by ANOVA with Dunnett’s post hoc test. (**h**) HCT116 cells or HT29 cells were treated with GW4869 at the indicated concentrations and were subjected to the soft-agar colony-formation assay. The mean numbers of colonies/cm^2^ is indicated. *p < 0.05, by ANOVA with Dunnett’s post hoc test. For all graphs, the error bars indicate the mean ± SD of three independent measurements. Uncropped gel images for panels b and f are shown in Supplementary Fig. 6.

To verify the role of exosome secretion in cancer cells, we inhibited exosome secretion in Src-transformed cells with the shRNA-mediated knock down of Rab27b, which is an essential component of exosome secretion^36^ (Fig. 6a). The Src activity was not inhibited by knockdown of Rab27b (Fig. 6b). In agreement with studies using several human cancer cells^37^, the downregulation of Rab27b substantially reduced exosome secretion; however, the restoration of shRNA-resistant Rab27b increased exosome secretion in Src–transformed cells (Fig. 6c). In our experimental conditions, the knockdown of Rab27b significantly suppressed colony-forming activity, whereas the rescue of Rab27b expression increased the colony-forming activity of Src–transformed cells (Fig. 6d). These results confirmed that exosome secretion is required for the maintenance of the transformed phenotype of cancer cells.

The role of exosome secretion was further evaluated using GW4869, an inhibitor of N-sphingomyelinase (nSMase) and a well-known inhibitor of exosome production^38^. GW4869 treatment inhibited exosome secretion in human cancer cells, including HCT116 and HT29 cells (Fig. 6e). GW4869 did not affect proliferation of these cells, however, GW4869 attenuated colony-forming activity in these cells in a dose-dependent manner (Fig. 6g,h). The expression and localization of Src/Alix were not changed by the knockdown of Rab27b (Fig. 6b and SupFig. 4b) or treatment of GW4869 (Fig. 6f and SupFig. 4c). Collectively, these results strongly suggest that exosome secretion plays a crucial role in the maintenance of tumor growth in some types of cancer cells.

## Discussion

**Figure 7:**
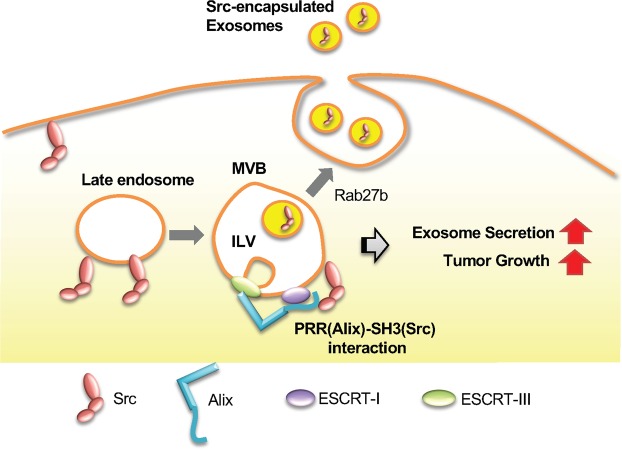
Hypothetical model of the Src/Alix interaction in regulating exosome biogenesis and tumor growth. When active c-Src localizes to the endosome membrane, c-Src interacts with Alix at the exosome membrane through the SH3 domain and promotes ESCRT-dependent ILV production, which results in the increase of exosome particles from cells. In turn, cancer cells maintain the transformed phenotype through the increased circulation and trafficking of cellular vesicles as exosomes.

In this study, we propose a mechanism by which Src–induced transformation promotes the secretion of exosomes, as shown in Fig. 7. In this model, activated c-Src in endosome membranes associates with Alix via a SH3-PRR interaction, which is inhibited by the closed conformation of inactivated c-Src. While this process may involve the phosphorylation of Alix by Src^39^, this modification is not important for the formation of exosomes (Fig. 3). Our findings indicate that Src–mediated activation of Alix promotes ILV formation in MVB, resulting in the promotion of exosome secretion observed in various human cancer cells with elevated Src activation.

Although there are many reports of the spatial distribution of c-Src, the relationship between the activity of c-Src and its intracellular localization are not well understood^40^. Since Src would have an affinity for lipid membranes through a myristoylation, and some Src molecules are found at endosome membranes whether Csk exists or not (SupFig. 4d), it is possible that the localization of c-Src is not strictly controlled by its activity. However, in this study, we show that activated c-Src tends to localize to the endosome membrane in Csk^−/−^ cells. While c-Src at focal adhesions plays a pivotal role in activating downstream signaling for cancer progression^41,42^, the function of c-Src at the endosome membrane has not been well established. We found that Src and Alix are localized at endosomes in tumor cells (HCT116) and non-tumor cells (HaCaT) (SupFig. 4e). In human cancer cells harboring Src activation, the localization of c-Src to the endosome membrane was not changed by Alix (SupFig. 4f), suggesting that c-Src intrinsically localizes to endosome membranes and active c-Src interacts with Alix on them. This interaction then triggers Alix-mediated ILV formation in MVB, which is required for the upregulation of exosome secretion. In addition, we demonstrated that activated c-Src in cancer cells is involved in the enhanced secretion of exosomes. Although the physiological significance of Src–mediated exosome secretion is currently under investigation, it is noteworthy that many molecules on the endosome membrane have been shown to transmit signals relating to various cellular events of biological importance^43^. Therefore, encapsulation of excess Src into MVB/ILV, and its evacuation in exosomes, may contribute to maintaining active levels of Src signaling. Indeed, we have often observed that aberrant expression of oncogenes, such as *v-src*, causes cell death in the early stage of cellular transformation (data not shown).

In this study, we also found that a constitutively active mutant of c-Src, SrcYF, showed superior activity for the promotion of exosome secretion compared with v-Src, despite both having comparably high kinase activity (Fig. 2). These results emphasize the importance of using wild-type c-Src (and its derivative) to study the role of Src in complicated cellular process regulated by protein-protein interactions. However, v-Src (with high transformation activity) has been used in many studies of Src–mediated oncogenic pathways^44^.

It has been suggested that exosomes assume roles in inter-cellular communication by transferring their cargo molecules, such as proteins and miRNAs. However, the reasons and mechanisms underlying the elevated secretion of exosomes often observed in various human cancer cells are not clear. We found that c-Src, which is known to be upregulated in various human cancers, is concentrated with its binding molecules and substrates in exosomes secreted from Src-induced transformed cells. It has been also reported that c-Src and its substrates are enriched in the exosomes secreted from prostate cancer cells^45^. Although these molecules possibly contribute in cancer phenotypes of recipient cells^46^, our findings led us to examine which molecule is responsible for exosome formation. From our experiments, Alix was identified among the candidates. Alix is believed to promote membrane scission around the necks of forming ILVs wrapped by ESCRT proteins^47^. Nevertheless, it remains to be clarified whether the upregulation of the Src–Alix axis in tumor cells is responsible for the elevated secretion of exosomes observed in human cancers.

Previously, it has been shown that Src phosphorylates Y319 of Alix^39^, and phosphorylated Alix can then recognize the SH2 domain of Src^26^. In this study, we found that c-Src directly binds to Alix in a phosphorylation-independent manner via a SH3-PRR interaction, which was proven to be necessary for Src-enhanced ILV formation. It should be noted that the Alix molecule has several atypical and typical PRR motifs consisting of many successive prolines at the C-terminal site; however, the stoichiometry and atomic details of the binding in these regions to Src SH3 remain elusive. Furthermore, we revealed that kinase activity is also necessary for the promotion of exosome secretion, suggesting that other Src substrates, such as Syntenin^32^, may be involved in this process.

In order to confirm that the association between Alix and activated c-Src promotes ILV formation, we attempted two different approaches. First, microscopic analysis using Rab5-active mutant–mediated endosome enlargement was performed, followed by biochemical analysis of the *in vitro* re-constitution of MVB. In *in vitro* ILV formation, we modified a reconstructed MVB system previously developed for the analysis of incorporated cargo in ILV^35^. We demonstrated that this method, using a loaded molecule as a probe (e.g., c-Src-Nluc^48^), would provide a novel tool for investigating the encapsulation process of exosome cargo molecules.

As mentioned above, our results indicate that the Src–Alix interaction is important to not only elevate exosome secretion but also to maintain the tumorigenicity of Src-transformed Csk^−/−^ fibroblasts and c-Src–activated human cancer cells. Intriguingly, the expression level of Alix in some tumor tissues (e.g., pancreatic cancer) shows a correlation to the prognosis (SupFig. 5a). Under our experimental conditions, in pancreatic cancer cells such as PANC-1 or MIAPaCa-2, Alix knockdown suppressed the tumor growth of each cell (SupFig. 5b). It has been reported that malignant alteration of pancreatic cancer increases exosome release^22^. Although more extensive research is needed using human cancer tissues and exosomes, our results support the possibility of Alix as a diagnostic biomarker of cancer progression.

Moreover, we also revealed that cell transformation inhibition by the reduction of exosome release is caused not only by the suppression of Alix but also by Rab27b, which is involved in vesicular fusion, and GW4869, which is a neutral sphingomyelinase inhibitor widely used for blocking exosome generation. Notably, these factors did not affect cell proliferation. These findings suggest that exosome secretion dysfunction can suppress cell transformation, regardless of cause. Considering that exosomes are secreted from normal cells also, this process may be important for facilitating intracellular vesicular traffic smoothly and to maintain cellular homeostasis by discarding excess substances in both normal and cancer cells. However, our results imply that the enhanced secretion of exosomes from cancer cells is likely driven by cancer-specific mechanisms, such as the Src–Alix interaction, and targeting this pathway may be an attractive therapeutic strategy that may serve to inhibit not only tumor progression but also cancer metastasis mediated by exosomes.

## Methods

### Chemicals and antibodies

Chemicals and antibodies used in this study included Alexa Fluor 488-phalloidin, HRP-conjugated goat anti-rabbit IgG and HRP-conjugated goat anti-mouse IgG (Thermo Fisher Scientific, Waltham, MA, USA); dasatinib (Sigma-Aldrich, St Louis, MO, USA); anti-phosphotyrosine 4G10, anti-Alix ABC40 and anti–v-Src ab-1 (Merck, Darmstadt, Germany); anti-Alix 3A9 and anti–phospho-Src pY418 (Cell Signaling Technology, Danvers, MA, USA); normal mouse IgG, anti-Csk C-20, anti-Tsg101 C-2 and anti-GAPDH 6C5 (Santa Cruz Biotechnology, Santa Cruz, CA, USA); anti-AnxA2 and anti-flotillin (BD Biosciences, San Jose, CA, USA); anti-CHMP4 (Abcam, Cambridge, UK); anti-Tubulin (Thermo Fisher Scientific).

### Cell culture, immunoblotting, immunoprecipitation and immunocytochemistry

Csk^−/−^ cells expressing Src genes, HT29 and HCT116 were cultured as described previously^49^. Immunoprecipitation and immunocytochemistry were performed as described previously^27,49^. In western blots of exosomes, samples prepared from cells were diluted with an equal volume of sample buffer. The intensity of bands is proportional to the concentration of each protein in an exosome particle and the number of secreted particles.

### Exosome preparation

The exosome producing culture was started from 1.5 × 10^6^ cells with 1% exosome-depleted FBS containing DMEM. After 48 hours of culture, the culture supernatant was harvested and final cell amounts were counted to confirm no growth differences arose among the different conditions or cells. Exosomes were prepared by ultracentrifugation^38^ with 25 mM trehalose to prevent the aggregation of exosomes^50^. The size, distribution, and concentration of the exosomes were determined using a NanoSight LM10 instrument (Malvern Panalytical, Malvern, UK).

### LC-MS/MS analysis

Exosomes were prepared from Csk^−/−^/c-Src-Flag cells, and Src-binding proteins were immunoprecipitated with the anti-Flag antibody. Immunoprecipitates were analyzed by LC-MS/MS as previously described^49^.

### Gene expression and shRNA

All gene-transfer experiments were carried out using pCX4 retroviral vectors^51^. v-Src was kindly provided by Dr. Tsuyoshi Akagi (Osaka Bioscience Institute) and GFP-human Rab5(Q79L) mutant was kindly provided by Dr. Hiroshi Hanafusa (Nagoya University). Mouse CD9, CD63, Rab5, Rab7, Rab11, human Rab27b, mouse Alix, mouse c-Src, c-Src conjugated with EGFP, mCherry, mScarlet, and NanoLuc, were PCR amplified and subcloned into the pCX4 vector. Production of and infection with retroviral vectors were performed as previously described^51^. Lentiviral vectors, both empty and carrying mouse or human Alix (ID: NM_011052.1, ID: NM_013374.2) and mouse Rab27b (ID: NM_030554.2) were purchased from Sigma.

### Quantitative Real-Time PCR

Total RNA was prepared using Sepasol (Nacalai Tesque, Kyoto, Japan). Real-Time PCR analysis was performed as described^52^. The primers used were as follows: mouse Rab27b:5′-ACTCAGAGGAACCAGTGATGGAG-3′, 5′-ATAGATTGGGGTCAGGGGAGA-3′, mouse GAPDH: 5′-AAAATGGTGAAGGTCGGTGTG-3′, 5′-AATGAAGGGGTCGTTGATGG-3′.

### Soft-agar colony-formation assay

Soft agar colony formation assay was performed as described^13^. Single-cell suspensions of 4 × 10^4^ cells were plated in 6-well culture dishes in 1.5 ml of DMEM containing 10% FBS and 0.36% agar on a layer of 2.5 ml of the same medium containing 0.7% agar. Colonies were stained with 3-(4,5-dimethylthiazol-2-yl)-2,5-diphenyltetrazolium bromide (MTT) 6–14 days after plating, and micrographs were used to count the numbers of colonies.

### *In vitro* multivesicular body formation assay

This assay is based on methods developed by Shurtleff *et al*.^35^ with the exception of using *Oplophorus gracilirostris* luciferase, i.e., Nanoluc (Promega, Madison, WI, USA)^53^. For *in vitro* reconstitution, the complete reaction consisted of membrane solution, incorporation buffer, Nanoluc substrate, ATP regeneration system, and cytoplasm solution. The mixture was then incubated at 30 °C for 20 min to promote the MVB forming reaction. The mixture was centrifuged at 15,000 × g for 10 min at 4 °C and the supernatant was removed. The pellet was treated with trypsin for 1 h at 4 °C. The remaining protected luciferase activity as a result of MVB formation was measured.

### Statistical analysis

All summary data were reported as the means ± S.D. calculated for each group and compared using the Student’s *t*-test or ANOVA with Dunnett’s post hoc test using Excel software (Microsoft). Test results were reported as two-tailed *p*-values, where *p* < 0.05 was considered statistically significant.

## Supplementary information

Supplementary Figures

## Data Availability

All data generated or analyzed during this study are included in this published article.
