# Supplementary material for: Src in endosomal membranes promotes exosome secretion and tumor progression
Source: Sci Rep. 2019 Mar 1;9:3265. doi: 10.1038/s41598-019-39882-z (PMC6397170; doi:10.1038/s41598-019-39882-z)
Supplement: Supplementary file 1 — Supplementary Figures [file 41598_2019_39882_MOESM1_ESM.pdf]

Src in endosomal membranes promotes exosome secretion and tumor progression

†Tomoya Hikita[1], †Atsushi Kuwahara[1, 2], Risayo Watanabe[1], Mamiko Miyata[1] and *Chitose Oneyama[1, 2, 3]

[1] Division of Microbiology and Oncology, Aichi Cancer Center Research Institute, Nagoya, Japan.

[2] Department of Oncogene Research, Research Institute for Microbial Diseases, Suita, Osaka, Japan.

[3] JST, PRESTO, Nagoya, Japan.

Supplementary Figures

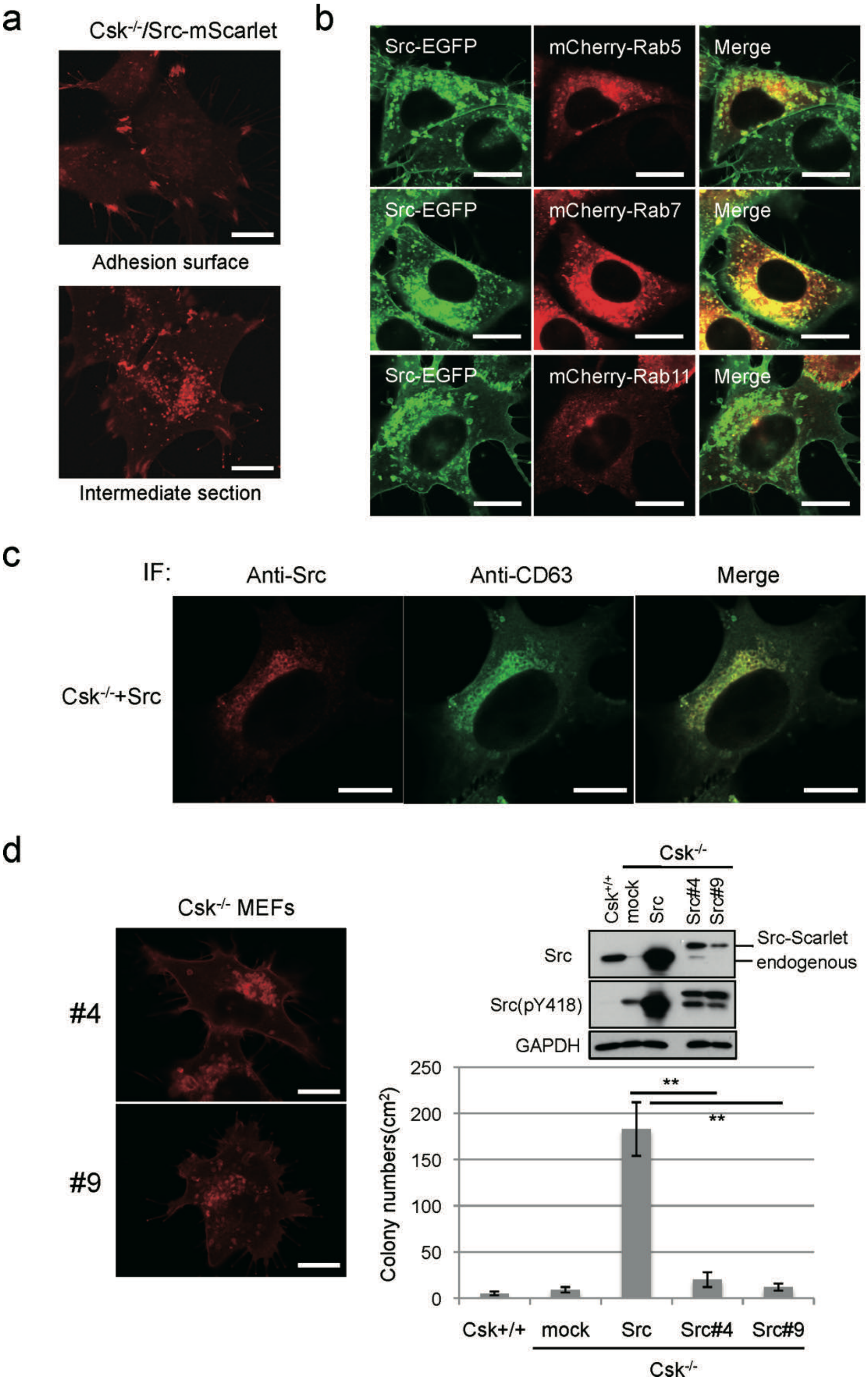

SupFigure 1. Active Src localizes to late endosome membranes in Src-transformed cells. (a) Confocal cross-sectional images of $Csk^{-/-}$ cells expressing mScarlet-conjugated Src (Src-mScarlet) at different depths. The localization of Src was shown at the adhesion surface (upper panel) and the intermediate section (lower panel). (b) EGFP-conjugated Src (Src-EGFP) and mCherry-conjugated Rab5, Rab7, or Rab11, were expressed in $Csk^{-/-}$ cells, and the localization of Src was analyzed. (c) The intracellular localization of Src in Src-expressing $Csk^{-/-}$ cells was analyzed by immunostaining with anti-Src or anti-CD63. (d) Cloned Src-mScarlet-expressing $Csk^{-/-}$ cells (Src#4 and 9) were generated (upper panels at the right), and soft-agar colony-formation activity (lower panel at the right) was analyzed. The mean number of colonies/cm^2 ± SD was obtained from three independent experiments. Under these conditions, the intracellular localization of Src in $Csk^{-/-}$+Src#4 and $Csk^{-/-}$+Src#9 was analyzed by confocal microscopy. Scale bar = 10 μm. $^{**}p < 0.01$, by ANOVA with Dunnett's post hoc analysis.

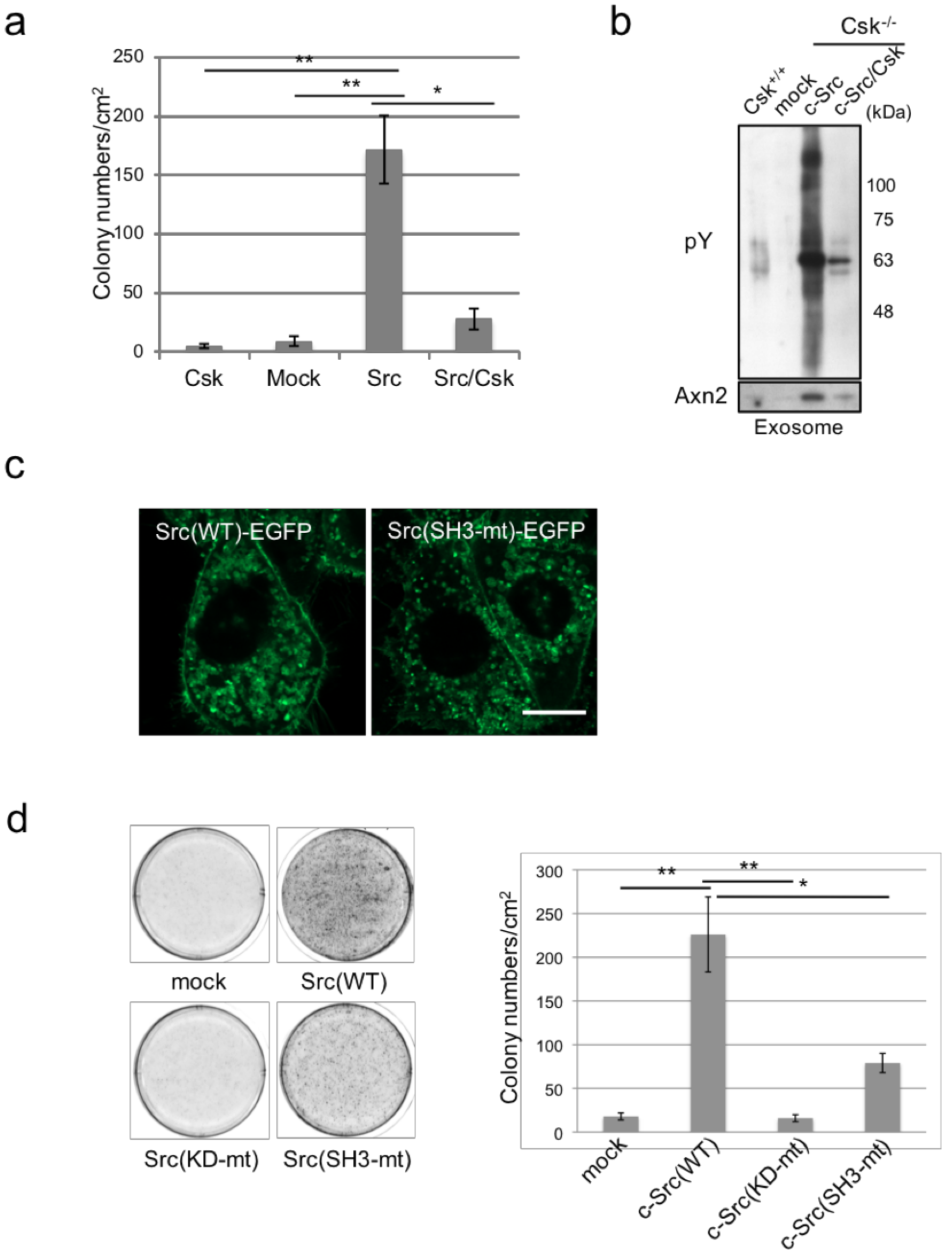

SupFigure 2. Transforming activity of Src. (a) Csk$^{+/+}$ cells and Csk$^{-/-}$ cells expressing mock, c-Src, or c-Src with Csk were subjected to the soft-agar colony-formation assay. (b) The exosome lysates from cells indicated in (a) were immunoblotted with the indicated antibodies. (c) EGFP-conjugated wild-type (SrcWT) or SH3-mutant (Src SH3mt) was expressed in Csk$^{-/-}$ cells, and the localization of Src was analyzed. Scale bar = 10 μm. (d) Csk$^{-/-}$ cells expressing the indicated constructs were subjected to the soft-agar colony-formation assay. Colonies were scored 7 days after plating. The mean number of colonies/cm^2 ± SD was obtained from three independent experiments. *$p < 0.05$ and **$p < 0.01$, by ANOVA with Dunnett's post hoc analysis.

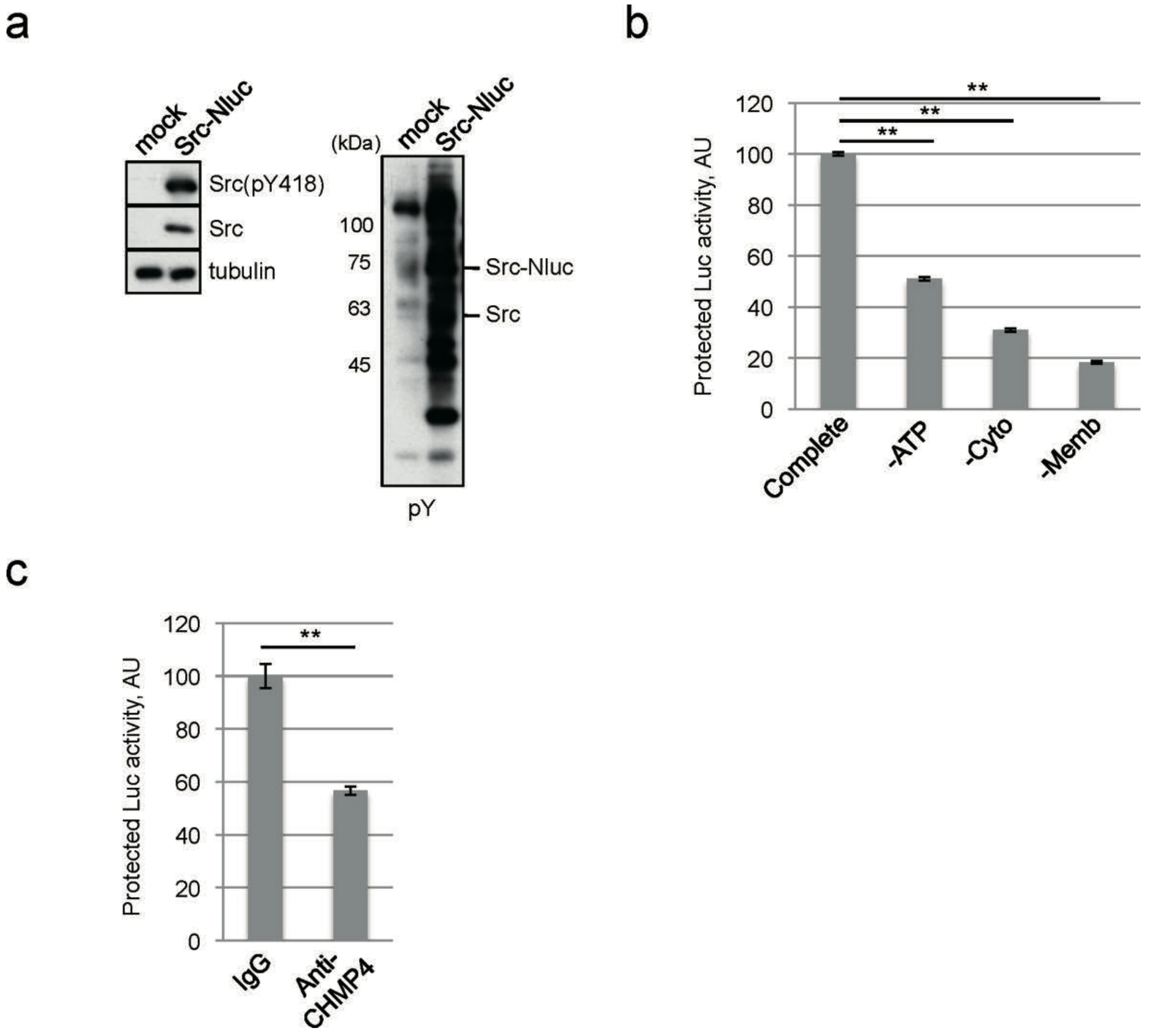

SupFigure 3. Construction of the *in vitro* MVB formation assay.

(a) Total cell lysates from $Csk^{-/-}$ cells expressing mock or Src conjugated with Nanoluc (Src-Nluc) were analyzed by immunoblotting with the indicated antibodies. (b) *In vitro* MVB formation by relative protected Src-Nluc. Reaction mixtures with or without ATP, cytosol, and membranes were incubated at 30°C for 20 min, and protected luciferase activity was measured. (c) The inhibitory effect of anti-CHMP4 as a neutralizing antibody for MVB formation. Reaction of cytosol and membranes from $Csk^{-/-}$ cells expressing Src-Nluc, with or without anti-CHMP4 neutralizing antibody, was performed and protected luciferase activity was measured. Results were normalized to the complete reaction or IgG control. For all graphs, the error bars indicate the mean ± SD of three independent experiments. $^{**}p < 0.01$, by ANOVA with Dunnett's post hoc analysis.

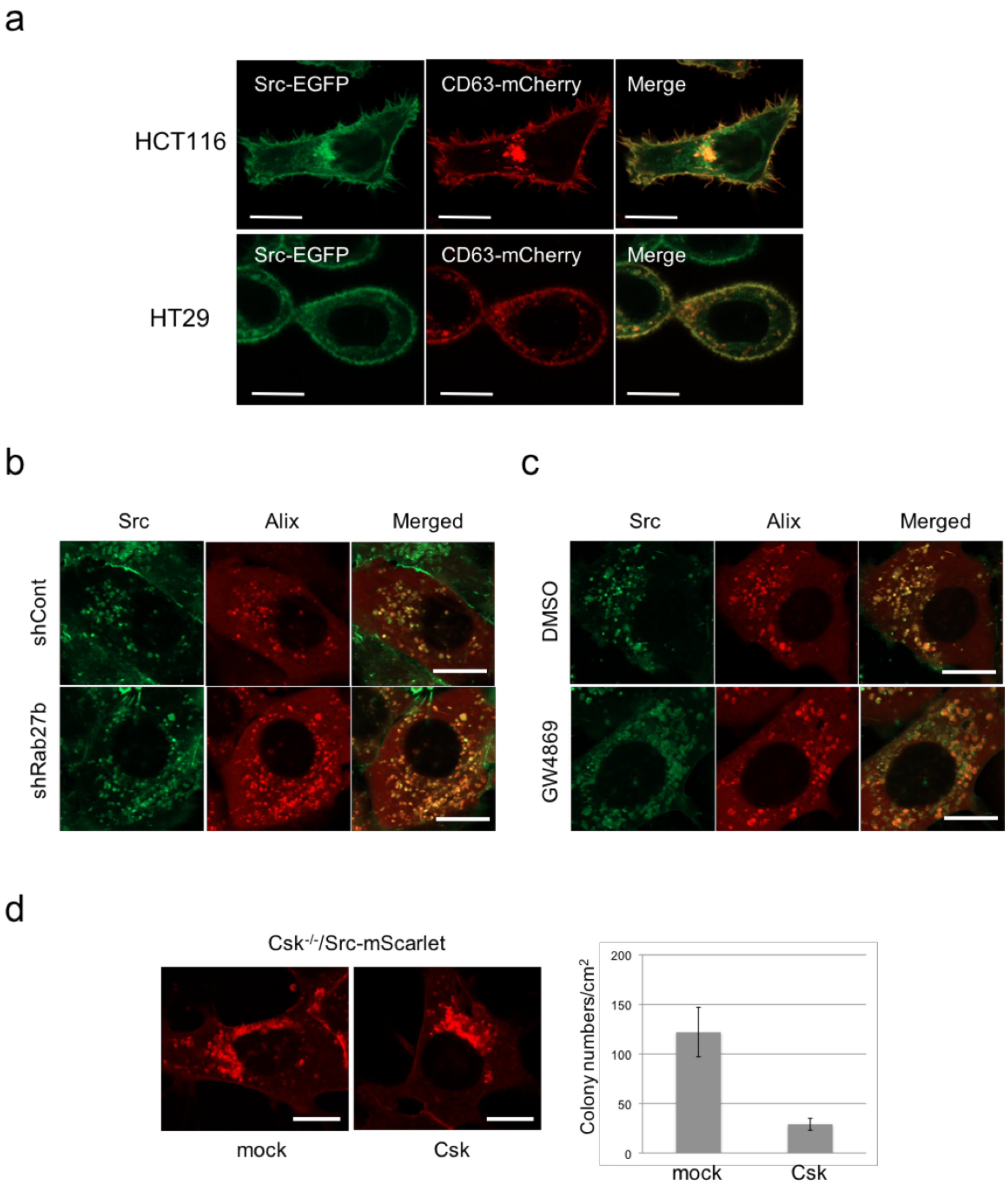
a
HCT116
HT29
Src-EGFP
CD63-mCherry
Merge
b
Src
Alix
Merged
shCont
shRab27b
c
Src
Alix
Merged
DMSO
GW4869
d
Csk-/-/Src-mScarlet
mock
Csk
Colony numbers/cm2
200
150
100
50
0
mock
Csk

e

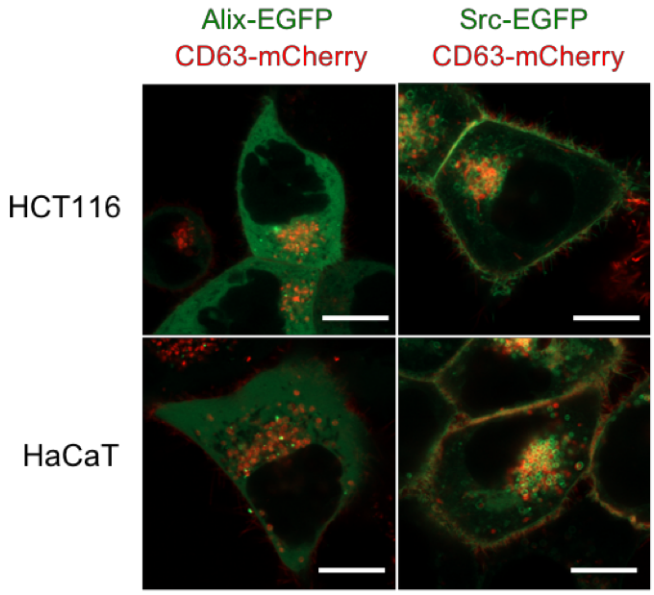

f

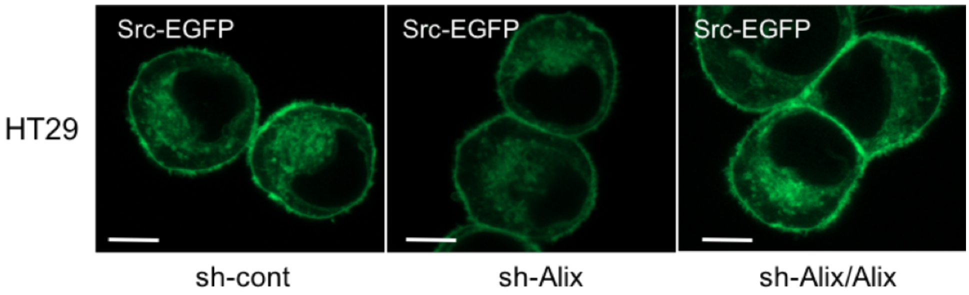

SupFigure 4. Src localizes to endosome membranes. (a) EGFP-conjugated Src (Src-EGFP; green) and mCherry-conjugated with CD63 (red) were expressed in HCT116 and HT29 cells, and the co-localization of Src with CD63 was analyzed. (b) EGFP-conjugated Src (green) and mCherry-conjugated Alix (red) were expressed in HCT116 cells expressing control (shCont) or Rab27b shRNA (shRab27b), and the co-localization of Src with Alix was analyzed. (c) HCT116 cells expressing EGFP-conjugated Src (green) and mCherry-conjugated Alix (red) were treated with GW4869 and the co-localization of Src and Alix was analyzed. (d) The localization of Src was analyzed in Csk$^{-/-}$ cells expressing Src-mScarlet with mock or Csk (left panels). These cells were subjected to soft-agar colony-formation assays and the mean number ± SD of colonies was obtained from three independent experiments (right panel). (e) mCherry-conjugated CD63 (red) with EGFP-conjugated Alix or Src (green) was expressed in HCT116 and HaCaT cells. The localization of Src and Alix in endosome was shown. (f) HT29 cells expressing control

(sh-cont) or Alix shRNA (sh-Alix), with or without sh-resistant mouse Alix, were introduced with Src-EGFP, and Src localization was analyzed by confocal microscopy. Scale bar = 10 μm.

a

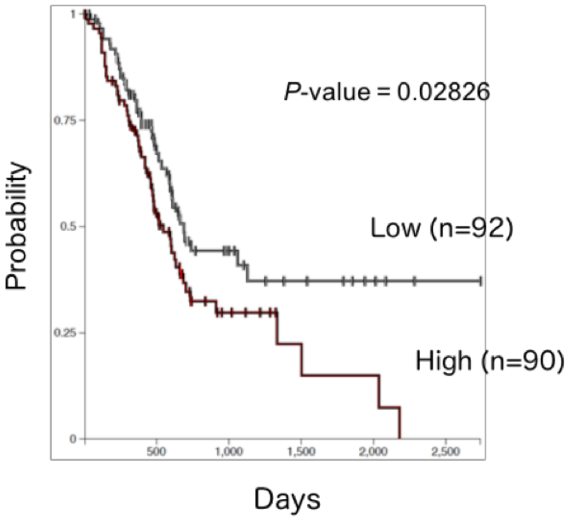

b

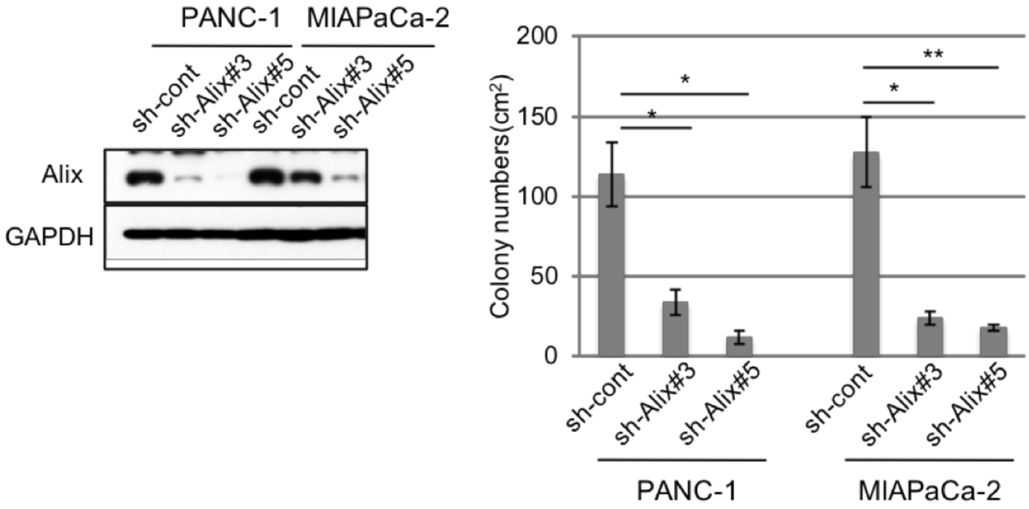

SupFigure 5. Alix in human pancreatic cancers. (a) The association of Alix expression with overall survival in pancreatic cancers from the Kaplan Meier plot, through the re-analysis of TCGA Pancreatic Cancer. (b) Total cell lysates from PANC-1 or MIAPaCa-2 cells expressing control (sh-cont) or Alix shRNA (sh-Alix) were analyzed by immunoblotting with the indicated antibodies (left panels). These cells were subjected to the soft-agar colony-formation assay. The mean number of colonies/cm^2 ± SD was obtained from three independent experiments. *$p < 0.05$ and **$p < 0.01$, by ANOVA with Dunnett's post hoc analysis.

Figure 1d

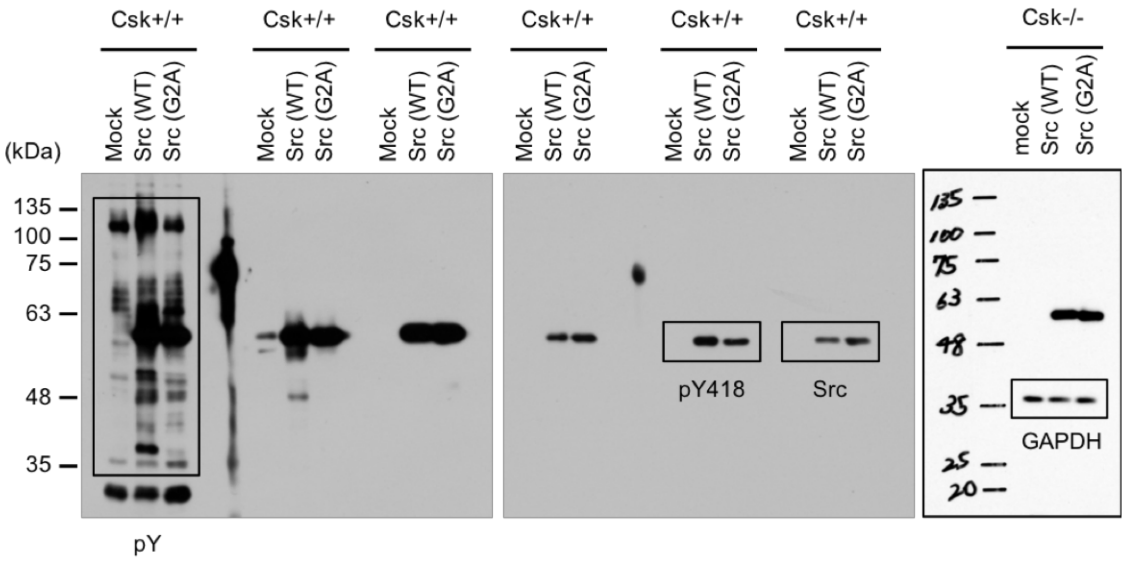

Figure 1f

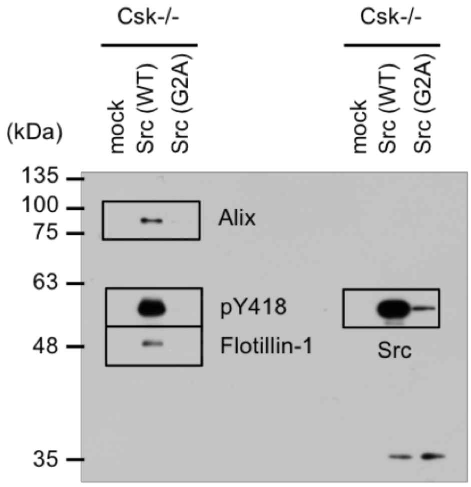

Figure 2b.

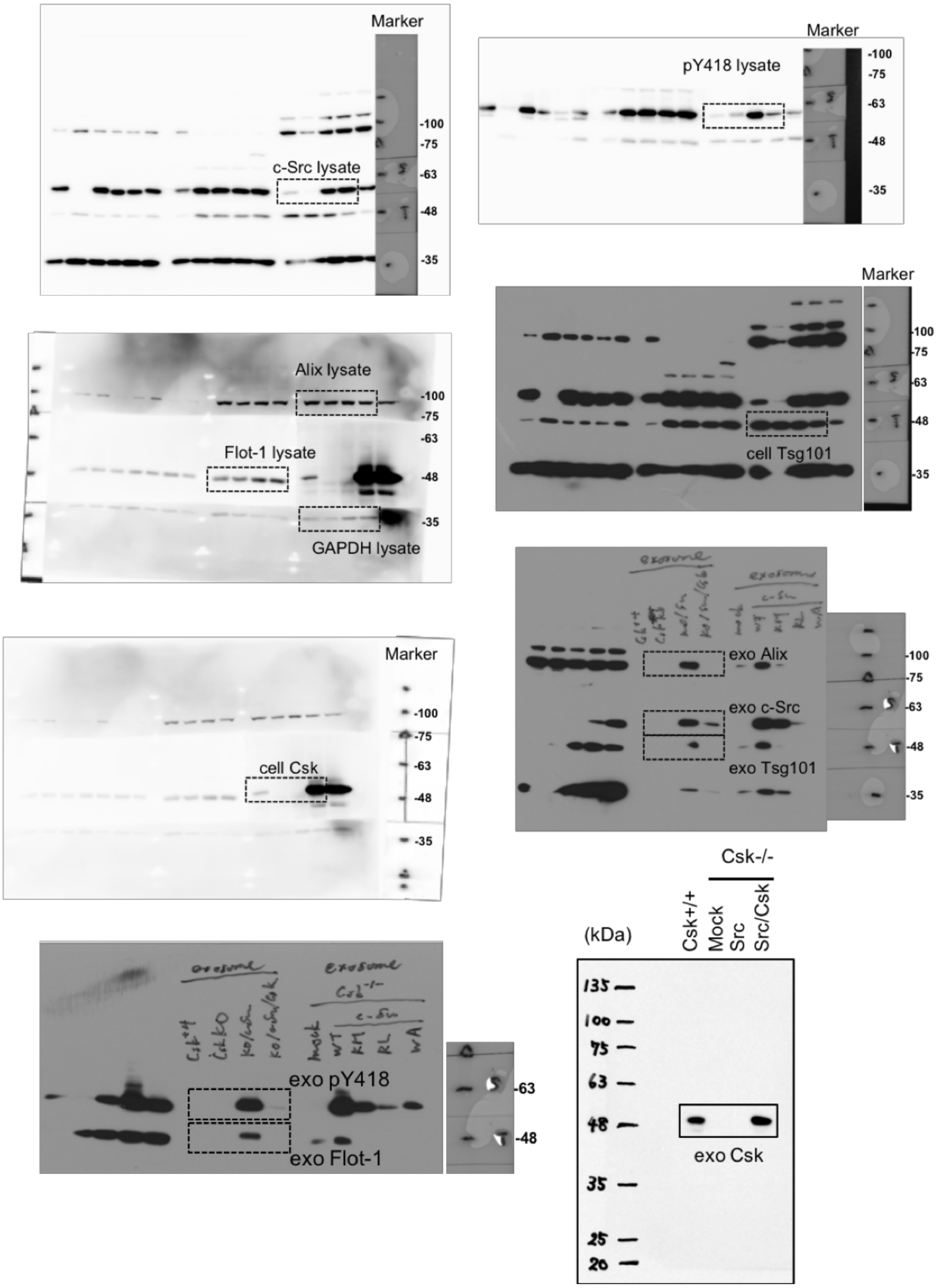
Marker
c-Src lysate
-100
-75
-63
-48
-35
pY418 lysate
Alix lysate
Flot-1 lysate
GAPDH lysate
cell Tsg101
cell Csk
exo Alix
exo c-Src
exo Tsg101
exo pY418
exo Flot-1
Csk-/-
(kDa)
Csk+/+
Mock
Src
Src/Csk
135
100
75
63
48
35
25
20
exo Csk

Figure 2c

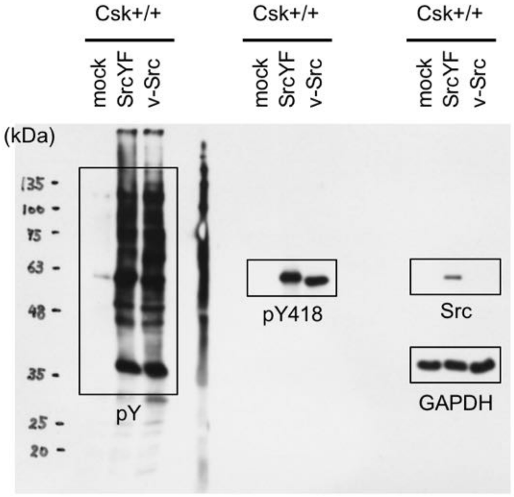

Figure 2d

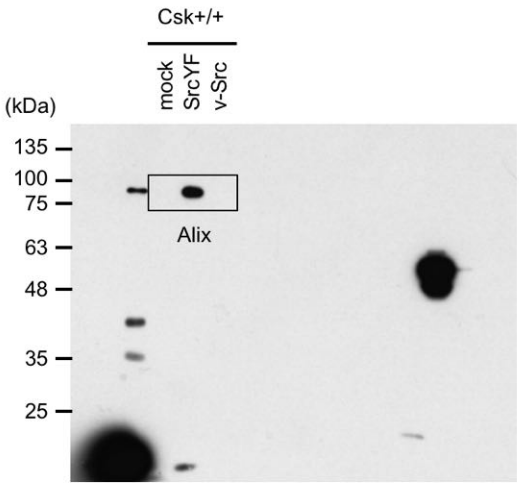

Figure 2d

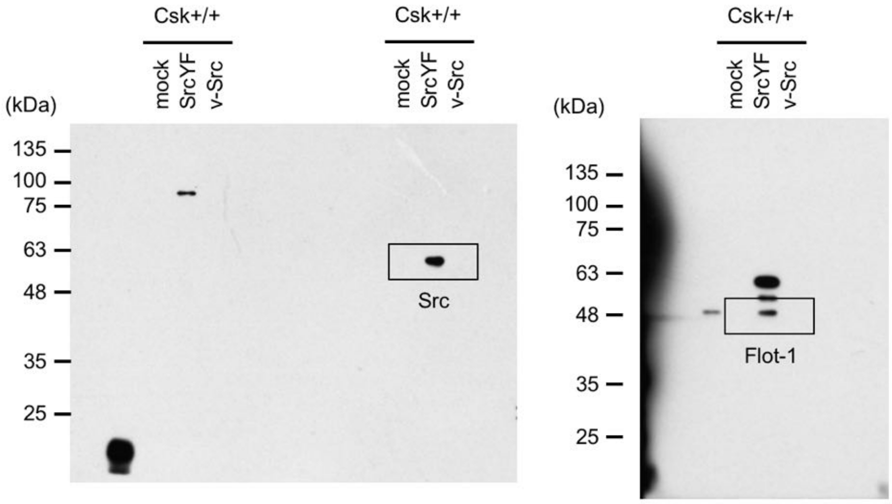

Figure 2e

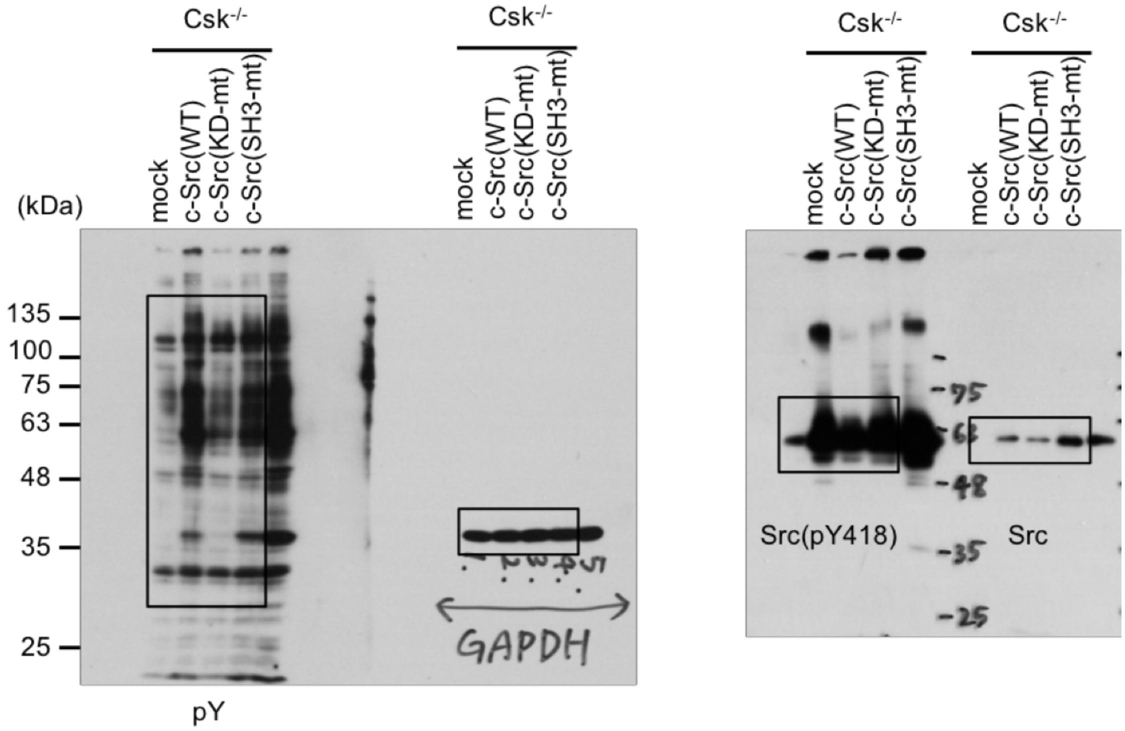

Csk-/-
mock
c-Src(WT)
c-Src(KD-mt)
c-Src(SH3-mt)
(kDa)
135
100
75
63
48
35
25
pY
GAPDH
Src(pY418)
Src

Figure 3a

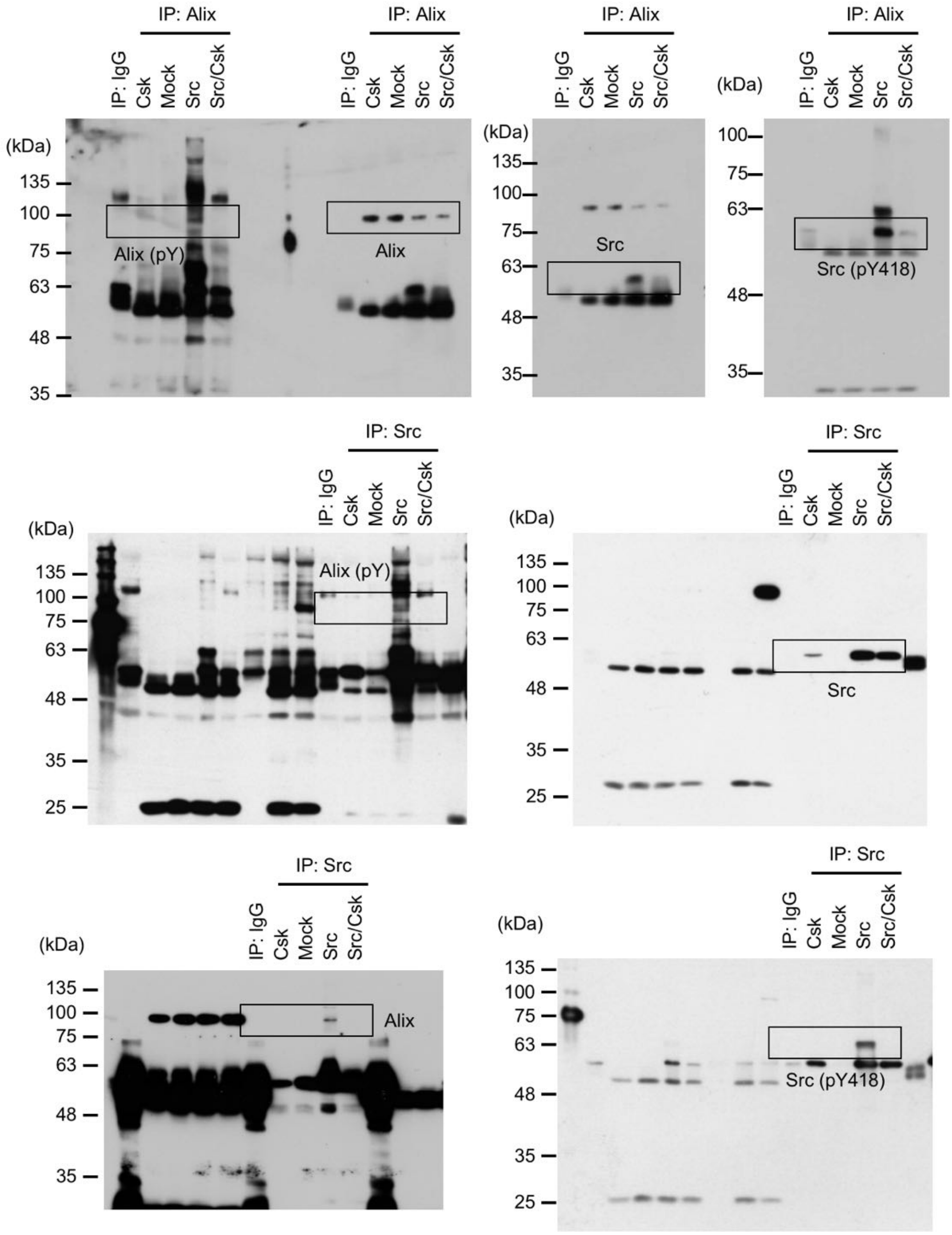
IP: Alix
IP: IgG
Csk
Mock
Src
Src/Csk
(kDa)
135
100
75
63
48
35
25
Alix (pY)
Alix
Src
Src (pY418)
IP: Src

Figure 3c

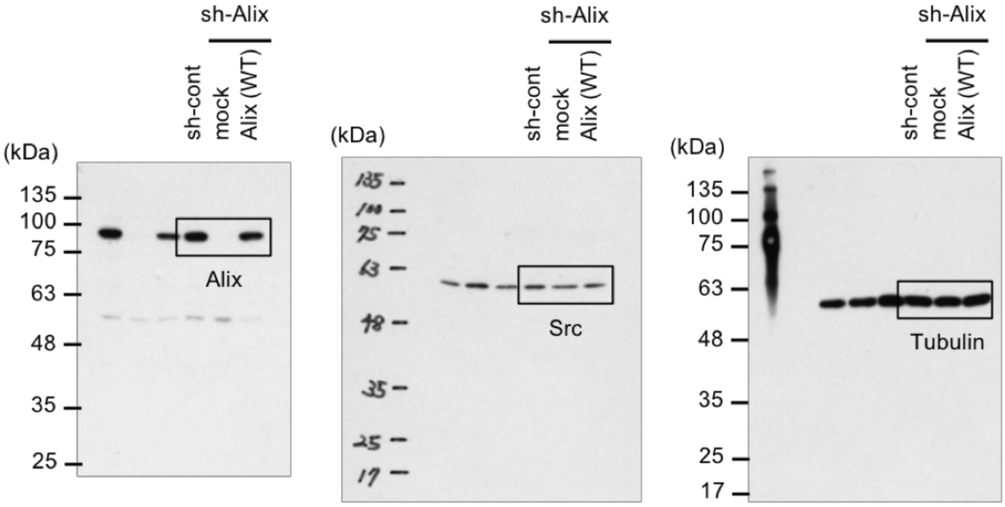

Figure 3g

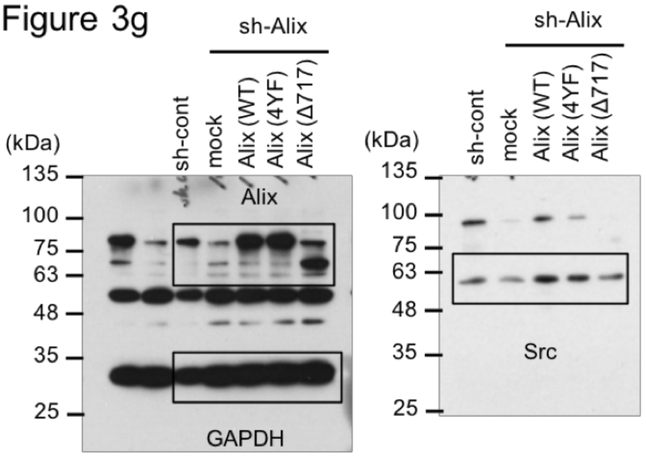

Figure 3h

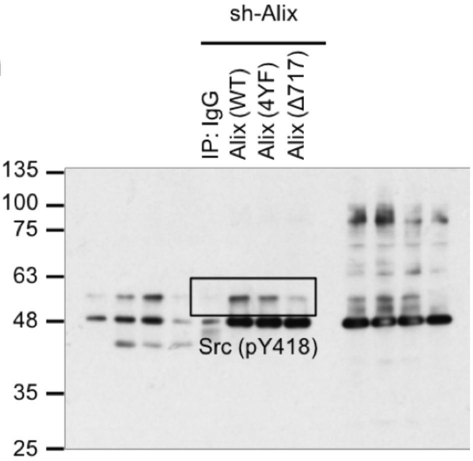

Figure 5a

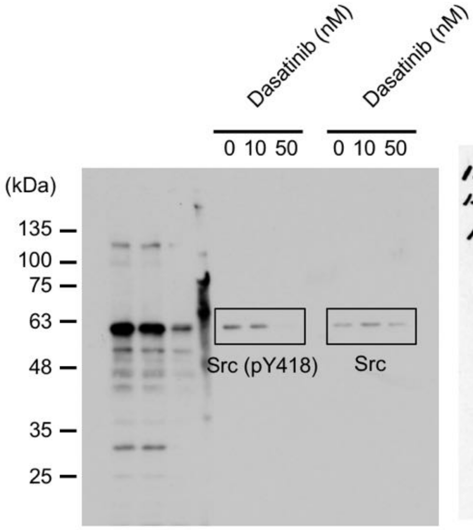

Figure 5c

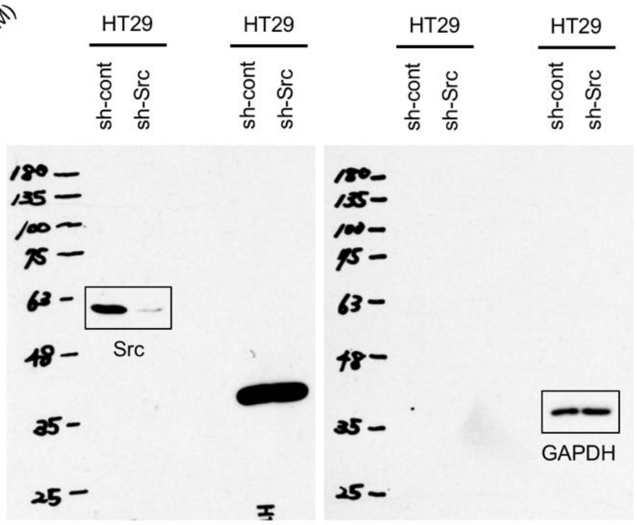

Figure 5e

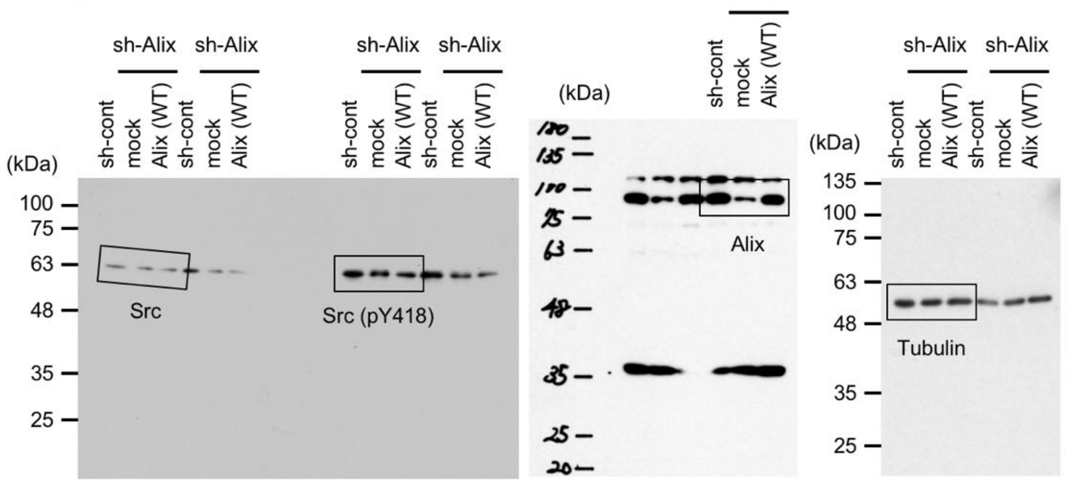

Figure 6b

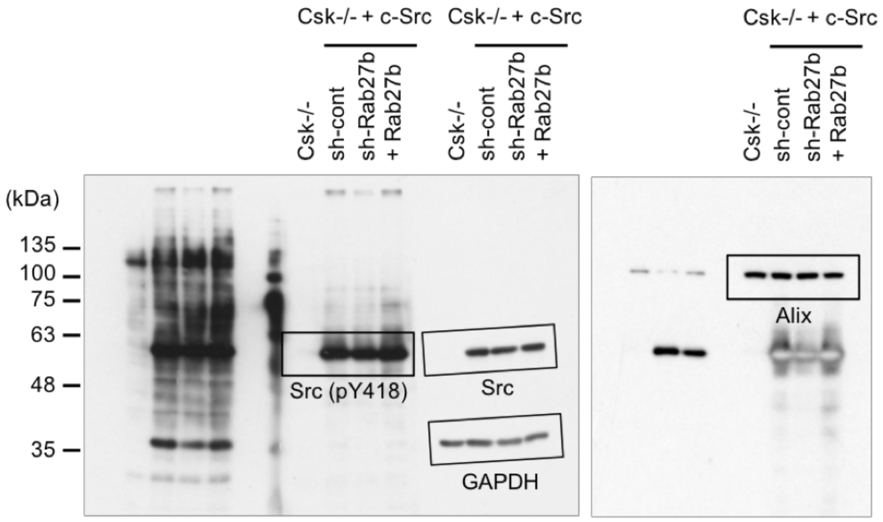

Figure 6f

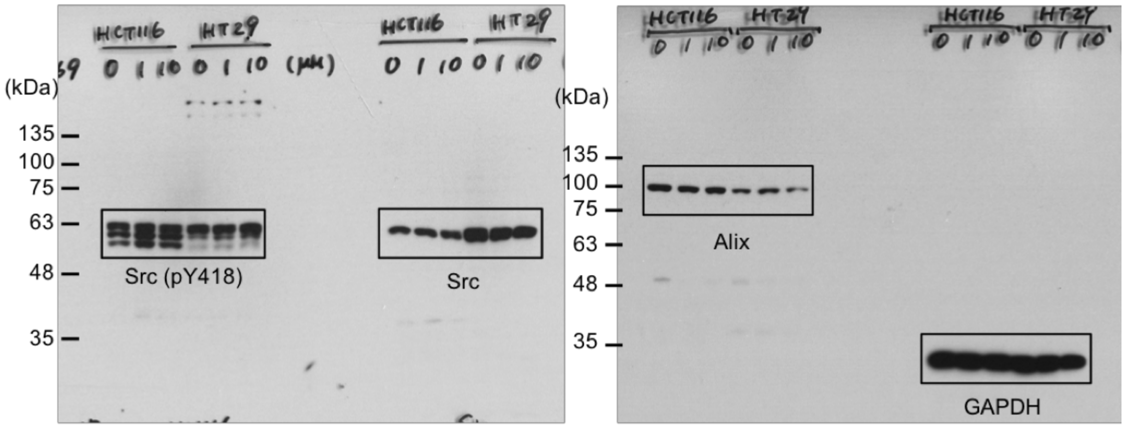

Supl Figure 1d

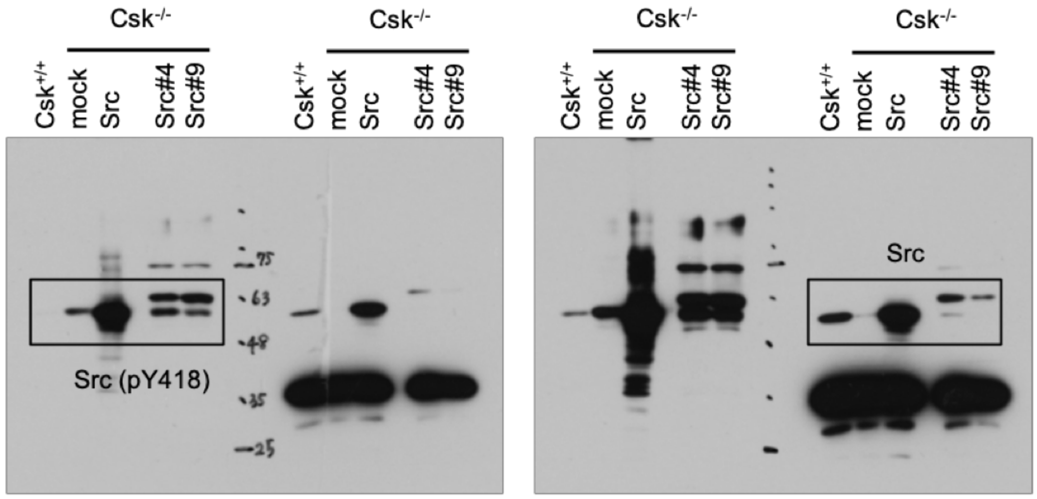

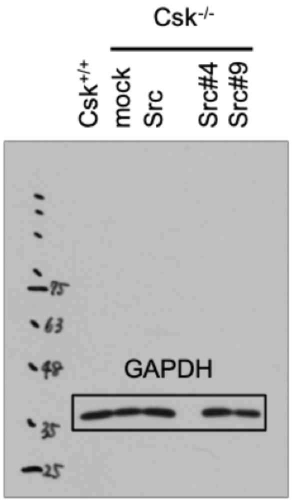

Supl Figure 2b

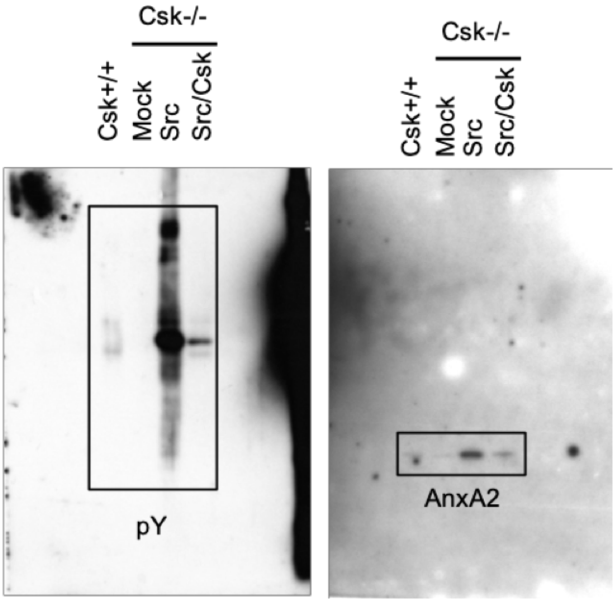

Supl Figure 3a

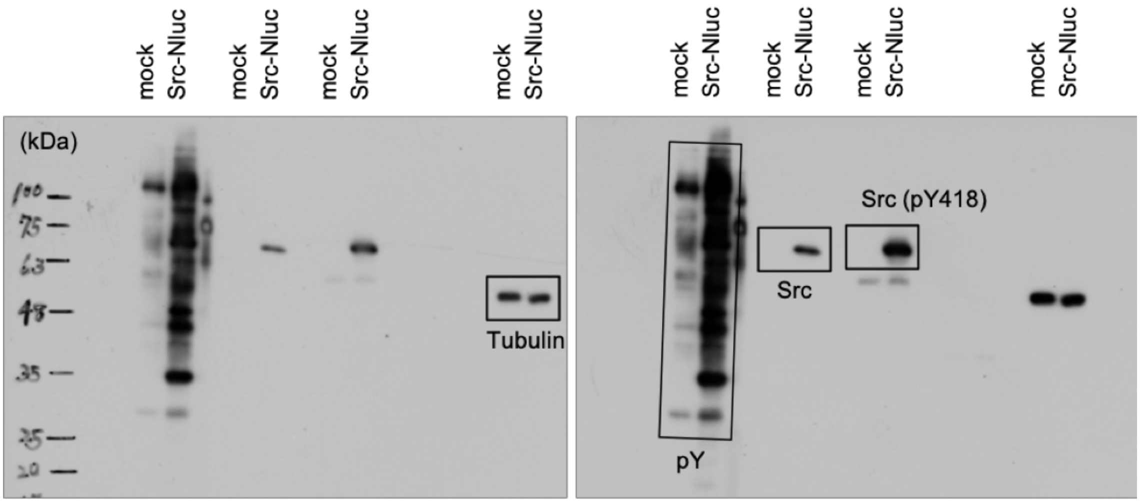

Supl Figure 5b

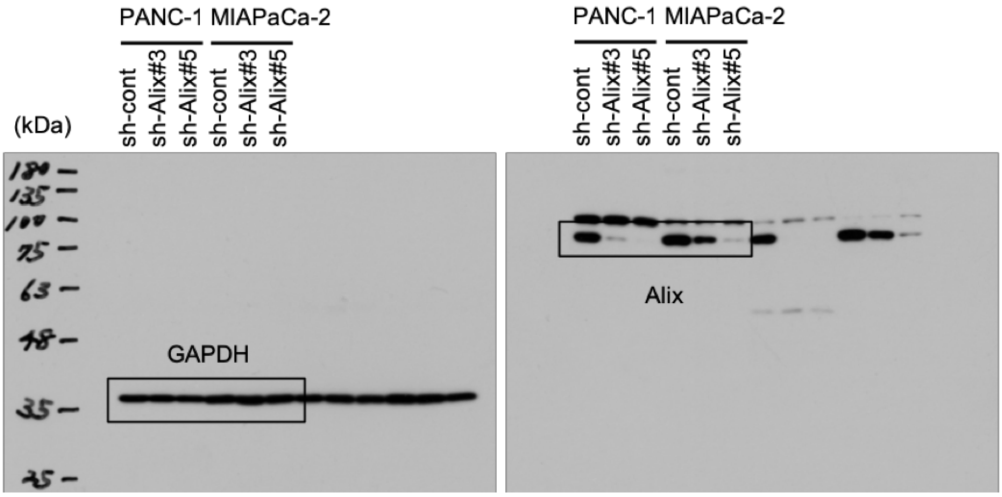

SupFigure 6.

Full scan images of Figures.
